# Oxidative stress-driven disease-associated microglia (DAM)-like polarization in human microglia (HMC3) cells exposed to small-size silver nanoparticles in a transwell co-culture system with neurons (cholinergic differentiated SH-SY5Y) cells in vitro

**DOI:** 10.1007/s00204-025-04183-0

**Published:** 2025-09-17

**Authors:** Bartosz Skóra, Konrad A. Szychowski

**Affiliations:** Department of Biotechnology and Cell Biology, Medical College, University of Information Technology and Management in Rzeszow, St. Sucharskiego 2, 35-225 Rzeszow, Poland

**Keywords:** Silver nanoparticles, Inflammation, Neurodegeneration, Disease-associated microglia, Neurons

## Abstract

Silver nanoparticles (AgNPs) are well-established pro-oxidative and pro-inflammatory factors, with the strongest effects observed in smaller NPs. This is particularly important due to the increasing accumulation of AgNPs in the environment and the human food chain in recent years. This accumulation has been correlated with an increased number of neurodegenerative diseases, in which microglia-driven inflammation plays a pivotal role. Importantly, the disease-associated microglia (DAM) phenotype has gained special attention. Therefore, this study aimed to determine the role of small-size AgNPs in inducing a DAM-like phenotype in a transwell co-culture model of human microglia (HMC3) and neurons (differentiated SH-SY5Y) in vitro. Our results demonstrated time- and dose-dependent cytotoxicity of small-size AgNPs in both tested cell models. Moreover, in the transwell co-culture system of HMC3 and differentiated SH-SY5Y cells, exposure to AgNPs led to ROS-dependent inflammation, associated with TLR4 and NF-κB activation, subsequently upregulating DAM-related proteins (e.g., ICAM1, ITGAX, ApoE, and B2M). A time-dependent increase in ^•^NO levels was also observed. Notably, reduced SYN1 and SNAP-25 expression, along with increased CAT, SOD, TRAF6, and TLR4 expression in differentiated SH-SY5Y cells, indicates ROS- and inflammation-mediated neuronal dysfunction. These findings indicate that the use of small-size AgNPs should be reconsidered due to their potential neurotoxic effects, but further investigation is necessary in future, using in vivo models.

**Supplementary Information:**

The online version contains supplementary material available at 10.1007/s00204-025-04183-0.

## Introduction

Silver nanoparticles (AgNPs) are among the most commonly used nanostructures today due to their high biological activity, particularly their antibacterial properties, even against resistant bacterial strains [as reviewed by Fernandes et al. (Fernandes et al. 2023)]. Consequently, increasing amounts of these nanoparticles have been detected in river water, sediments, and fish tissues, suggesting their potential accumulation within the human food chain (Ihtisham et al. 2021; Mat Lazim et al. 2023). A recent study presented by Babaei et al. (2022) confirmed the bioaccumulation potential of AgNPs along the phytoplankton–zooplankton–fish axis, with bioaccumulation factors (BCFs) reaching approximately 826, 131, and 1000, respectively (Babaei et al. 2022). These findings are significant given the well-documented cytotoxic properties of AgNPs in normal cells, such as human keratinocytes (HaCaT), patient-derived normal human fibroblasts, and intestinal epithelial cells (Franková et al. 2016; Carrola et al. 2016; Gokulan et al. 2020). Notably, numerous studies have demonstrated a size-dependent toxicity of AgNPs, with smaller nanoparticles exhibiting greater toxic effects (Kim et al. 2012; Perde-Schrepler et al. 2019). The induction of oxidative stress—manifested as elevated reactive oxygen species (ROS) levels, modulation of antioxidant enzyme activity, and DNA integrity disruption—has been identified as a key mechanism of AgNPs toxicity (Nallanthighal et al. 2017; Mikhailova 2020). Similar effects have been observed in cells derived from the nervous systems of humans and animals, including rat and human embryonic stem cells, mouse microglial (N9) cell lines, and rat-derived astrocytes (Liu et al. 2015; Dan et al. 2015; Gonzalez-Carter et al. 2017). However, data regarding the effects of AgNPs on microglial cells remain inconsistent.

For instance, Sharma et al. demonstrated that green-synthesized AgNPs (using honeyberry extract) induced a protective M2-like phenotype shift in lipopolysaccharide (LPS)-stimulated human-derived microglial cells (HMC3) (Sharma et al. 2023). This effect may be attributable to the antioxidant properties of the honeyberry-derived extract used as a capping agent for nanoparticle synthesis (An et al. 2020). Conversely, Shang et al. reported that AgNPs impaired the autophagy process in mouse microglial cells (BV2), leading to their pro-inflammatory polarization (Shang et al. 2022). Furthermore, 10 nm AgNPs demonstrated cytotoxicity in a mouse-derived co-culture model comprising BV2 cells, astrocytes (ALT), and differentiated neurons (N2a), resulting in apoptosis, necrosis, and neurodegeneration (Hsiao et al. 2017). In recent years, a growing body of research has linked neurodegenerative diseases, such as Parkinson’s disease (PD), Alzheimer’s disease (AD), and dementia to the specific polarization of microglial cells, known as disease-associated microglia (DAM) (Deczkowska et al. 2018; Gao et al. 2023). This phenotype is typically associated with fluctuations in the activity of the triggering receptor expressed on myeloid cells 2 (TREM2) during a two-stage process. The first stage (TREM2-dependent) involves increased TREM2 activity, suppression of microglia-specific genes (e.g., *CX3CR1, P2RY12, TMEM119, HEXB, Cst3*), and upregulation of cytoskeleton- and metabolism-related genes (e.g., *APOE, B2M, TYROBP*). The second stage (TREM2-independent) is characterized by elevated mRNA expression of genes, such as *ITGAX*, *AXL*, and *CD9*, though knowledge in this area is still evolving (Deczkowska et al. 2018). This phenomenon is particularly concerning given the increasing environmental presence of AgNPs and their ability to bioaccumulate in the food chain, posing potential risks to human health. Importantly, Xiong et al. demonstrated that AgNPs can cross the blood–brain barrier (BBB) in rats, while Trickler et al. showed that AgNPs induce an inflammation-like phenotype in BBB-derived cells, underscoring the need to evaluate the effects of small-size AgNPs on brain-derived cells (Tang et al. 2008; Trickler et al. 2010). Although some studies, such as those by Hsiao et al., have investigated the cytotoxic properties of 10 nm AgNPs in co-culture models of mouse microglia, astrocytes, and neuron-like cells, further research is required, particularly in the context of DAM-related proteins and human-derived cells (Hsiao et al. 2017).

The present study aims to assess the ability of small-size AgNPs to induce a DAM-like phenotype in human-derived cell models in vitro, with a focus on the role of NF-κB in this process. Additionally, a developed transwell co-culture model of human neurons (cholinergic-differentiated SH-SY5Y cells) and human microglial cells (HMC3) was employed to better simulate cell–cell interactions in the nervous system and extrapolate the obtained results.

## Materials and methods

### Reagents

The fetal bovine serum (FBS), fetal bovine serum heat inactivated (FBS HI), Universal RNA purification Kit, radioimmunoprecipitation assay (RIPA) buffer, Fast Probe qPCR Master Mix, and Perfect^™^ Tricolor Protein were purchased from EURx (Gdańsk, Poland). The phosphate-buffer saline (PBS), DMEM, and DMEM/F12 media were obtained from Corning Life Sciences (Corning, NY, USA). The penicillin/streptomycin, trypsin, all-trans retinoic acid (RA), resazurin sodium salt, acrylamide/bisacrylamide, sodium chloride (NaCl), 4-methyl-N1-(3-phenylpropyl)−1,2-benzenediamine (JSH-23, NF-κB inhibitor), lipopolysaccharide from *E. coli* (LPS), N-acetyl-L-cysteine (NAC), bovine serum albumin (BSA), 2′,7′-dichlorodihydrofluorescein diacetate (H_2_DCF-DA), 4-amino-5-methylamino-2’,7’-difluorofluorescein diacetate (DAF-FM), glycine, Tris–HCl, Tris-Base, hydrochloric acid (HCl), immobilon-P (pore size: 0.45 µm), Tween-20, Triton-X100, paraformaldehyde, goat serum, yellow/green fluorescence latex beads, Millicell^®^ 6-well hanging cell culture inserts, and PVP-coated AgNPs with 5 nm size were purchased from MERCK (Rahway, NJ, USA). The culture inserts compatible with 12-well plates with 0.4 µm pore size (cat. 36012) was purchased from SPL Life Sciences (Pochon, Kyonggi-do, South Korea). The primary anti-TLR4 antibodies were kindly gifted by Proteintech (Rosemont, IL, USA). The lyophilized human brain-derived neurotrophic factor (BDNF) was purchased from R&D Systems (Minneapolis, MN, USA).

The results of the physicochemical characterization of AgNPs were presented previously (Skóra et al. 2022). No signs of agglomeration were observed during the experiments. The AgNPs solution was always sonicated (*A* = 30%, 10 min, RT) prior to being added to the cells. JSH-23 and LPS were purchased in powdered form, dissolved in sterile DMSO, and added to the cells by diluting them in the appropriate medium (1:1000); therefore, the volume of DMSO did not exceed 0.1%.

### Cell culture, differentiation, and treatment

The human embryonic microglial cells (HMC3, CRL-3304) and human neuroblastoma cells (SH-SY5Y, CRL-2266) were purchased from the American Type Culture Collection (Manassas, VA, USA). HMC3 cells were cultured in DMEM with 10% FBS and 0.1% penicillin/streptomycin, while SH-SY5Y cells were grown in DMEM/F12 with 10% FBS HI and 0.1% penicillin/streptomycin at 37 °C and 5% CO_2_. After reaching confluency, the cells were detached using trypsin and seeded into appropriate culture vessels (as described below).

#### Mono-culture-based experiments

**Fig. 1:**
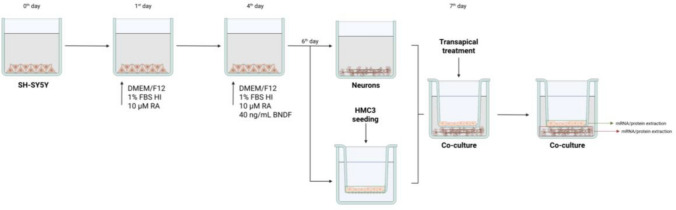
The scheme of the experimental design, using the developed transwell co-culture model of differentiated SH-SY5Y cells (model of neurons) and HMC3 cells (model of microglia). *FBS HI* fetal bovine serum heat inactivated, *RA* all-trans retinoic acid, *BDNF* brain-derived neurotrophic factor

The SH-SY5Y cells were seeded at a density of 4.5 × 10^3^ cells per well in a 96-well plate [for resazurin reduction, lactate dehydrogenase (LDH) release level, intracellular ROS level], or 8 × 10^4^ cells/chamber in the eight-chamber culture slides, using a standard culture medium. The cholinergic differentiation of the cells was performed according to the previously published protocol (Skóra and Szychowski 2025). Briefly, after 24 h (D1), the differentiation procedure was initiated by treating the cells with Differentiation Medium #1 (DMEM/F12 containing 1% FBS HI and 10 µM RA); see Fig. 1. After 72 h (D4), the medium was discarded and replaced with Differentiation Medium #2 (DMEM/F12 containing 1% FBS HI, 10 µM RA, and 40 ng/mL BDNF); see Fig. 1. After another 72 h (D7), the differentiation process was completed, and the cells were treated or co-treated with a medium containing tested compounds (as described in each subsection) and/or conditioned medium.

HMC3 cells were seeded at a density of 4 × 10^3^ cells per well for 24-h or 48-h treatments and 3× 10^3^ cells per well for the 72-h treatment in the 96-well plates [for resazurin reduction, LDH release level, intracellular ROS level, nitric oxide (^•^NO) generation assays], 5 × 10^5^ cells/dish in the ⌀60 mm culture dish (for phagocytosis assay), or 5 × 10^4^ cells/chamber in the 8-chamber culture slides. The cells were allowed to attach for 24 h. After this period, the medium was removed and replaced with fresh medium-containing tested compounds (as described in each subsection).

#### Transwell co-culture-based experiments

SH-SY5Y cells were seeded in the basolateral chambers of 12-well plates (for RT-qPCR) or 6-well plates (for Western Blot) at densities of 2 × 10^5^ and 3.5 × 10^5^ cells/well, respectively, and subcultured for 24 h. Differentiation was then induced (as described in subsection "Mono-culture-based experiments") and continued until day 7 (Fig. 1). On the 6th day of differentiation, HMC3 cells were seeded separately in the apical chambers of 12-well, or 6-well culture inserts for RT-qPCR and Western Blot, respectively, at densities of 1.5 × 10^5^ cells/well (12-well) or 2.5 × 10^5^ cells/well (6-well). Cells were cultured separately for 24 h. On the 7th day of differentiation, the inserts were transferred into wells, containing neurons (Fig. 1). At the same time, fresh medium containing 1 µg/mL AgNPs, 10 µM JSH-23, or 4 mM NAC—either individually or in co-treatment—was added to the apical chamber for 24 h (RT-qPCR) or 72 h (Western Blot). The conditioned medium (CM) was collected under sterile conditions, frozen at −80 °C, and used for immunofluorescence analysis. The overall experimental setup is illustrated (Fig. 1).

### Resazurin reduction assay and LDH release level

The resazurin reduction method and LDH release level assay were performed as described previously (Skóra et al. 2022, 2024a). Briefly, cells were exposed to AgNPs at concentrations ranging from 1 ng/mL to 100 µg/mL in the respective medium (DMEM with 10% FBS for HMC3 cells or DMEM/F12 with 1% FBS HI for neurons) for 24, 48, or 72 h. In co-treatment experiments, cells were treated with 1 µg/mL AgNPs, 10 µM JSH-23, and 4 mM NAC, either individually or in co-treatment, for 24 h. Following the respective treatment periods, absorbance was measured at 490 nm for the LDH release assay, while fluorescence was recorded at an excitation wavelength of 570 nm and an emission wavelength of 590 nm. The measurements were taken, using microplate reader (Molecular Devices, FilterMax F5). Results were expressed as percentages (%) relative to control cells (vehicle-treated) for the corresponding treatment duration.

### ^•^NO generation

The ^•^NO generation assay was performed as described by Szychowski et al. (Szychowski and Gmiński 2019). Briefly, 24 h after seeding HMC3 cells in 96-well plates, the medium was removed and replaced with serum-free medium containing 5 µM DAF-FM. The cells were incubated for 30 min, after which the medium was discarded, followed by two washes with warm PBS. Fresh medium containing 1 µg/mL AgNPs, 10 µM JSH-23, or 4 mM NAC—either individually or in co-treatment—was then added for 3, 6, 24, or 48 h. After each time point, fluorescence intensity was measured using a microplate reader (Molecular Devices, FilterMax F5) at an excitation wavelength of 485 nm and an emission wavelength of 535 nm. Results were expressed as a percentage (%) of the control (vehicle-treated cells).

### Intracellular ROS level

The method was performed as described in a previous study without modifications (Skóra et al. 2024b). Cells were treated with AgNPs at concentrations ranging from 1 ng/mL to 100 µg/mL for 24 to 72 h, or with 1 µg/mL AgNPs, 10 µM JSH-23, or 4 mM NAC—either individually or in co-treatment—for 24 h. After the designated incubation period, fluorescence intensity was measured using an excitation wavelength of 485 nm and an emission wavelength of 535 nm. Results were expressed as percentages (%) relative to the control (vehicle-treated cells).

### Real-time qPCR

The method was performed as described by Szychowski and Skóra (Szychowski and Skóra 2024). Total RNA was isolated separately from the apical (HMC3) and basolateral (differentiated SH-SY5Y cells) chambers using the Universal RNA Isolation Kit (EURx, Gdańsk, Poland). The RNA concentration was measured and standardized for each sample (1400 ng for HMC3 and 700 ng for differentiated SH-SY5Y). Subsequently, reverse transcription was performed to obtain cDNA template, using random primers-containing kit, i.e., High-Capacity cDNA Reverse Transcription Kit (Thermo Fisher, Waltham, USA). RT-qPCR was conducted using specific TaqMan probes and primers targeting the following genes: *NFKB1* (Hs00765730_m1), *TREM2* (Hs00219132_m1), *IL1B* (Hs01555411_g1), *ApoE* (Hs04194724_g1), and *B2M* (Hs06637353_s1). The threshold cycle (Ct) value for each sample was calculated during the exponential phase, and the ΔΔ_Ct_ method was used to determine the average fold (Avg. Fold) expression of the target genes. *GAPDH* (Hs02758991_g1) and *RPS28* (Hs02597258_g1) were used as reference genes for differentiated SH-SY5Y and HMC3 cells, respectively.

### Western blot

The Western Blot method was performed as described in a previous study without modifications (Skóra et al. 2023a). Protein extraction was conducted 72 h after treatment, separately for the apical (HMC3; microglia model) and basolateral (differentiated SH-SY5Y; neuron model) chambers, using RIPA buffer. Fifty micrograms of protein were loaded onto the gel. GAPDH was consistently used as a loading control after stripping and reprobing the PVDF membranes with the appropriate buffer. The dilutions and manufacturers of the specific antibodies used in this study are presented in Table 1.

**Table 1:** The primary and HRP-conjugated antibodies used in this study

Primary antibodies			Secondary (HRP-conjugated) antibodies		
Target (host species)	Dilution	Producer/Cat. number	Target (host species)	Dilution	Producer/Cat. number
ICAM1 (Rb)	1:1000	ABClonal/A20472	Anti-Rb HRP-conjugated (Go)	1:2000	ThermoFisher/#31460
IκBα (Rb)	1:1000	ABClonal/A19714			
AXL (Rb)	1:1000	ABClonal/A22378			
MyD88 (Rb)	1:1000	ABClonal/A16889			
B2M (Rb)	1:4000	ABClonal/A11642			
ApoE (Rb)	1:1000	ABClonal/A0304			
ITGAX (Rb)	1:1000	ABClonal/A1508			
PPARγ (Rb)	1:1500	ABClonal/A11183			
IBA1 (Rb)	1:1000	ABClonal/A19776			
CAT (Rb)	1:1000	ABClonal/A11780			
SOD2 (Rb)	1:1000	ABClonal/A19576			
MAP2 (Rb)	1:1000	ABClonal/A22206			
ROCK1 (Rb)	1:2000	ABClonal/A11158			
TRAF6 (Rb)	1:1000	ABClonal/A23385			
SYN1 (Rb)	1:1000	ABClonal/A5247			
SNAP-25 (Rb)	1:1000	ABClonal/A0986			
SYP (Rb)	1:1500	ABClonal/A6344			
TLR4 (Rb)	1:1000	Proteintech/30400–1-AP			
SOD1 (Mo)	1:570	SCTB/sc-101523	Anti-Mo HRP-conjugated (Go)	1:3000	ThermoFisher/#31430
KI67 (Mo)	1:400	SCTB/sc-23900			
NF-κB (Mo)	1:3000	ABClonal/A10609			
GAPDH (Mo)	1:100 000	ABClonal/AC033			

The producers, catalog numbers and the applied dilution was also showed

*Rb* rabbit, *Mo* mouse, *Go* goat, *HRP* horseradish peroxidase

### Immunofluorescence

HMC3 cells were seeded in eight-chamber culture slides (SPL Life Sciences, cat. S30118) at a density of 5 × 10^4^ cells/chamber. After 24 h, the medium was removed and replaced with fresh medium-containing 1 µg/mL AgNPs, 10 µM JSH-23, 4 mM NAC, 1 µg/mL LPS, AgNPs/JSH-23, AgNPs/NAC, or AgNPs/LPS. These specimens were subsequently used in the first set of immunofluorescence (IF) staining (IF#1), consisting of TLR4-FITC/NF-κB-AlexaFluor^594^/DAPI.

SH-SY5Y cells were seeded at a density of 8 × 10^4^ cells/chamber in 8-well culture chamber slides and initially subcultured for 24 h. Afterward, the differentiation protocol was performed as described in subsection "Mono-culture-based experiments" (Fig. 1). On the 7th day of differentiation, the medium was discarded, and the conditioned medium (CM) from HMC3 treatment (containing 1 µg/mL AgNPs, 10 µM JSH-23, 4 mM NAC, 1 µg/mL LPS, AgNPs/JSH-23, AgNPs/NAC, or AgNPs/LPS) was applied for 24 h. These specimens were subsequently used in the second set of IF staining (IF#2), consisting of NF-κB-FITC/Tau-AlexaFluor^594^/DAPI.

After the designated incubation time, the cells were washed twice with PBS, fixed with 4% paraformaldehyde for 20 min at RT, and washed again twice with PBS to remove any fixation residues. The cells were then permeabilized using 0.1% Triton X-100 in PBS for 15 min on ice and washed three times with TBST. Next, the slides were blocked with 5% goat serum for 1 h at RT, and specific primary antibodies were added to the specimens (for IF#1 and IF#2: mouse anti-NF-κB—cat. A10609, ABClonal, dilution 1:100). After 1 h of incubation at RT, the slides were washed four times with TBST, and the appropriate secondary antibodies were applied for 1 h at RT (for IF#1: goat AlexaFluor^594^-conjugated anti-mouse—cat. AS077, ABClonal, dilution 1:100; for IF#2: goat FITC-conjugated anti-mouse—cat. AS001, ABClonal, dilution 1:100). After washing with TBST, the cells were blocked again with 5% goat serum for 30 min at RT. Rabbit primary antibodies were then added and incubated for 1 h at RT (for IF#1: rabbit anti-TLR4—cat. 30400–1-A, Proteintech, dilution 1:100; for IF#2: rabbit anti-Tau—cat. 46687T, Cell Signaling Technology, dilution 1:75). The slides were then washed three times with TBST, followed by the application of specific secondary antibodies (for IF#1: goat FITC-conjugated anti-rabbit—cat. AS011, ABClonal, dilution 1:100; for IF#2: goat AlexaFluor^594^-conjugated anti-rabbit—cat. AS039, ABClonal, dilution 1:100). After 1 h at RT, the cells were washed twice with TBST, and the nuclei were stained with 0.3 µg/mL DAPI for 5 min. The cells were then washed, mounted, and visualized using a confocal microscope (ZEISS LSM700) with blue, red, and green channels. Imaging was performed using 100× (ZEISS EC Plan-Neofluar 10 ×/0.30), 200 × (ZEISS Plan-Apochromat 20x/0.8 M27), and 630× (ZEISS Plan-Apochromat 63x/1.40 Oil DIC M27) objectives. Pseudocolors were applied using ImageJ software. Corrected Total Cell Fluorescence (CTCF) and fluorescence intensity plots were obtained using built-in ImageJ plugins.

### Phagocytosis assay: flow cytometry analysis and visualization

The phagocytosis assay was performed as described elsewhere, with modifications (Huang et al. 2016). In brief, HMC3 cells were seeded at a density of 5 × 10^5^ cells per dish in a ⌀60 mm culture dish and incubated for 24 h. After this period, the medium was removed and replaced with fresh medium containing 1 µg/mL of AgNPs, 4 mM NAC, 1 µg/mL LPS, or a co-treatment for either 24 or 72 h. On the day of analysis, the fluorescence of yellow–green latex beads (cat. L1030, MERCK) was prepared according to the manufacturer’s instructions. Specifically, the beads were opsonized in FBS (1:5, *v/v*) at 37 °C for 1 h. The resulting mixture was then diluted in DMEM at a 1:1000 ratio (*v/v*). After the designated treatment period, the medium was discarded, and the cells were exposed to the diluted latex beads for 2 h at 37°C. Next, the medium was removed, and the cells were washed five times with PBS to eliminate residual beads from the cell surface. The cells were then collected by trypsinization and centrifugation (500 × g, 5 min). The pellet was washed once with PBS, centrifuged again, and resuspended in cold PBS on ice. Shortly after, the phagocytic activity of the cells was measured using flow cytometry (BS Accuri C6 Plus) with an FL-1 detector. Autofluorescence was assessed using unstained cells (without beads), and a total of 15,000 cells were analyzed per sample. Results were expressed as the percentage of cells within the phagocytic population, compared to the control. The gating strategy was presented in Supplementary File.

To visualize latex beads uptake, HMC3 cells were seeded onto eight-chamber culture slides at the density of 5 × 10^4^ cells/chamber 24 h before the experiment. The cells were then treated as described above for 24 h, and the phagocytosis assay was conducted as previously described. However, following the washing steps, the cells were fixed with 4% paraformaldehyde for 15 min, washed three times with TBST, and permeabilized with 0.1% Triton X-100 in PBS at 4 °C for 15 min. Immunostaining was then performed as described in subsection "Immunofluorescence" using primary antibodies against β-actin (ACTB, cat. sc-47778; dilution 1:100; Santa Cruz Biotechnology) and secondary AlexaFluor^594^-conjugated goat anti-mouse antibodies (cat. AS077; dilution 1:100; ABClonal). The nuclei were stained with 0.3 µg/mL DAPI. Observations were conducted using a ZEISS LSM700 confocal microscope at 630 × magnification (Plan-Apochromat 63 ×/1.40 Oil DIC M27).

### Statistical analysis

The results are presented as means ± standard deviation (SD) from at least three independent experiments (*n* ≥ 3). Data were analyzed using a one-way analysis of variance (ANOVA) followed by Tukey’s post hoc test, with statistical significance indicated as *, **, and ***, corresponding to *p* < 0.05, *p* < 0.01, and *p* < 0.001, respectively. Additionally, a t-test was used to assess statistical differences between respective groups, with significance denoted as #, ##, and ### for *p* < 0.05, *p* < 0.01, and *p* < 0.001, respectively. The IC_50_ curves and *R*^2^ values were calculated based on the resazurin reduction assay results using a three-parameter compound vs. response curve.

## Results and discussion

In this paper, we have chosen two cell types, i.e., HMC3 cells, which are a well-established, immortalized microglial cell line (a model of human microglia), as well as RA/BDNF-based differentiated SH-SY5Y cells (a simplified model of human neurons). According to the literature, their phenotype makes them appropriate for use in in vitro screening studies (Dello Russo et al. 2018; de Medeiros et al. 2019). Furthermore, a significant increase (by 67%) in the MAP2 protein expression in cells differentiated for 7 days, compared to the non-differentiated SH-SY5Y proves the efficiency of the applied protocol due to the similar expression of this protein in mature neurons (Soltani et al. 2005) (Fig. 6F).

### Dose-, time-, and ROS-dependent effect of small-size AgNPs in human models of microglia and neurons in vitro

**Fig. 2:**
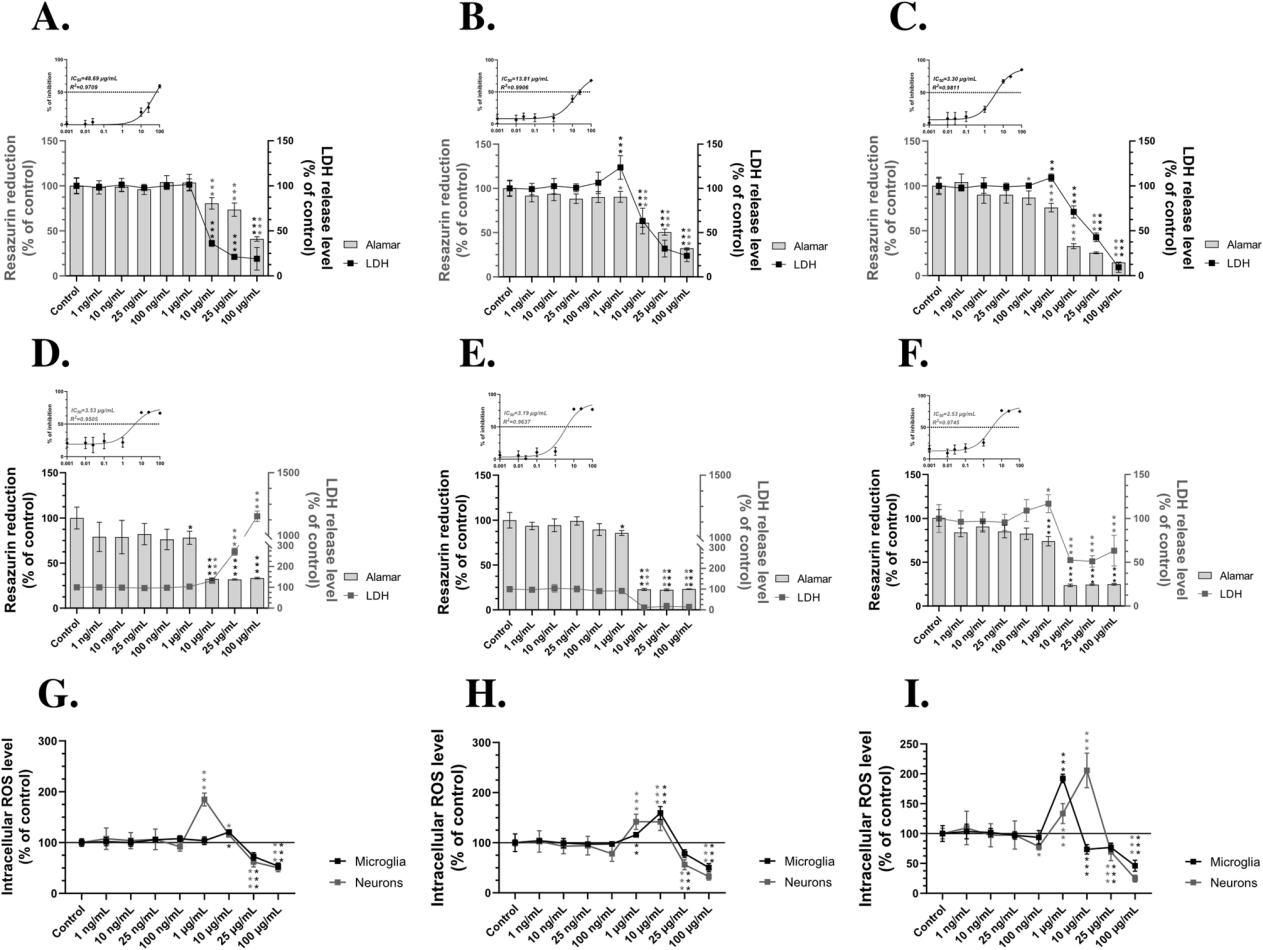
Dose- and time-dependent pro-oxidative mechanism of action of AgNPs. Metabolic activity (bars; Alamar), LDH release levels (lines), IC_50_ curves (insets) (**A**–**F**), and intracellular ROS levels (**G**–**I**) after treating microglia (HMC3, **A**–**C**, **G**–**I**) and neurons (differentiated SH-SY5Y cells, **D**–**F**, **G**–**I**) with AgNPs at concentrations ranging from 1 ng/mL to 100 µg/mL for 24 h (**A**, **D**, **G**), 48 h (**B**, **E**, **H**), and 72 h (**C**, **F**, **I**). The IC_50_ curves and *R*^2^ values were calculated based on the resazurin reduction assay results using a three-parameter compound *vs*. response curve. Data are presented as means ± SD (*n* = 6 for all presented data). Statistically significant differences compared to the control are denoted as *, **, and *** for *p* < 0.05, *p* < 0.01, and *p* < 0.001, respectively. All data presented in this figure were derived from mono-culture-based experiments

In the first part of our study, we conducted analysis, using a monoculture of microglia and neuron cellular models. To assess cytotoxicity, we employed two complementary methods, i.e., the resazurin reduction assay, which measures cell metabolic activity, and the LDH release assay, which indicates cell membrane integrity and cytotoxicity as well (Weidmann et al. 1995; Vieira-da-Silva and Castanho 2023). Our results demonstrate a time- and dose-dependent effect of small-size AgNPs in both tested cell models. In HMC3 cells, exposure to AgNPs resulted in a significant reduction in metabolic activity at concentrations ranging from 10 to 100 µg/mL across all tested time points, with decreases ranging from 19.41 to 83.31% compared to the control (Fig. 2A–C). A corresponding increase in LDH release was also observed at these concentrations, ranging from 63.81% to a maximum of 90.67% compared to the control (Fig. 2A–C). Notably, after 48 h and 72 h of exposure, even 1 µg/mL AgNPs’ concentration significantly reduced metabolic activity by 9.63% and 24.17%, respectively, compared to the control (Fig. 2B, C). At the same time points, LDH release levels for 1 µg/mL of AgNPs increased by 23.68% and 9.22%, respectively (Fig. 2B, C). The IC_50_ decreased over time, reaching 48.69 µg/mL (*R*^2^ = 0.9709) at 24 h, 13.81 µg/mL (*R*^2^ = 0.9906) at 48 h, and 3.30 µg/mL (*R*^2^ = 0.9811) at 72 h (Fig. 2A–C). Similarly, in differentiated SH-SY5Y cells, a significant decrease in metabolic activity was observed at AgNPs concentrations ranging from 1 µg/mL to 100 µg/mL at all tested time points (Fig. 2D–F). Interestingly, after 24 h of exposure, a significant increase in LDH release was observed, with values reaching 31.59% at 10 µg/mL, 171.17% at 25 µg/mL, and 954.82% at 100 µg/mL (Fig. 2D). However, at 48 h and 72 h, LDH release levels significantly decreased (Fig. 2E, F). The IC_50_ values remained relatively stable over time, reaching 3.53 µg/mL at 24 h (*R*^2^ = 0.9505), 3.19 µg/mL at 48 h (*R*^2^ = 0.9637), and 2.53 µg/mL at 72 h (*R*^2^ = 0.9745) (Fig. 2D–F).

The highest toxicity was observed at microgram concentrations in both cell types, consistent with previously reported effects of AgNPs on SH-SY5Y cells (Park et al. 2017; Li et al. 2018; Yamaguchi et al. 2022). Importantly, although an increased amount of LDH in the medium is typically associated with the cytotoxicity of the tested compound, our results show the opposite trend at the highest concentrations. This discrepancy may be explained by the high toxicity of AgNPs, which reduces the number of viable cells and/or causes rapid release of large amounts of proteases into the medium following membrane damage. Such effects may interfere with LDH stability and its measurement. This interpretation is supported by the resazurin reduction assay and by the lack of changes in IC_50_ values over time. Importantly, a similar tendency has been observed in other xenobiotic-related studies, such as the findings presented by Szychowski et al. for triclosan (Szychowski et al. 2019). Conversely, our findings contrast with those of Sharma et al., who reported a protective effect of green-synthesized AgNPs (derived from honeyberry) in HMC3 cells at concentrations between 15.625 and 125 µg/mL (Sharma et al. 2023). Additionally, Gonzalez-Carter et al. demonstrated that AgNPs (49.7 ± 10.5 nm) exhibit anti-inflammatory properties in immortalized embryonic mouse microglia (N9) cell line (Gonzalez-Carter et al. 2017). These discrepancies may be attributed to differences in NPs size and capping agents used in our study *vs.* cited papers, specifically polyvinylpyrrolidone (PVP) *vs.* honeyberry-derived protein corona, respectively. Such factors can directly influence AgNPs uptake, thereby affecting their reactivity and biological impact. However, our results align with those of Sheng et al., who demonstrated a dose-dependent increase in LDH release in HMC3 cells exposed to microgram concentrations of AgNPs, further supporting our findings (Shang et al. 2022).

With observed cytotoxic effects of small-size AgNPs, we next assessed their impact on intracellular ROS levels in both microglia and neurons. Such approach is justified by numerous studies demonstrating the pro-oxidative properties of AgNPs (Kim et al. 2014; Flores‐López et al. 2019). Our results support these findings, indicating that the cytotoxic effects of small-size AgNPs are associated with increased ROS levels in both tested cell lines, though primarily at mild-toxic concentrations. Neurons exhibited a significant increase in intracellular ROS levels across all tested time points at AgNP concentrations ranging from 1 to 10 µg/mL, with increases ranging from 15.42 to 105.68% compared to the control (Fig. 2G–I). A similar trend was observed in HMC3 cells, where ROS levels increased in a time- and dose-dependent manner, reaching a maximum increase of 92.23% at 1 µg/mL after 72 h of treatment (Fig. 2G–I). Consistently, Dayem et al. reported that AgNPs significantly enhance ROS generation in undifferentiated SH-SY5Y cells (Dayem et al. 2014). Guo et al. demonstrated that low concentrations of AgNPs (2 µg/mL) induce ROS production, leading to antioxidant enzyme activation and impaired neurogenesis in rat-derived neural stem cells (NSCs) (Guo et al. 2017). Likewise, Shang et al. found that AgNPs negatively impact microglia, triggering their activation (Shang et al. 2022). However, contrasting results were reported by Hsiao et al., who found that AgNPs at 1 µg/mL and 3 µg/mL did not alter ROS levels in the mouse microglial BV-2 cell line (Hsiao et al. 2017). These discrepancies may be attributed to differences in cell origin (mouse vs. human in our study) and NP size, as smaller AgNPs, like those used in our study, are generally more toxic.

Interestingly, at the highest tested concentrations (25–100 µg/mL), a significant decrease in ROS levels was observed between 24 and 72 h, reaching reductions of up to 75.40% in SH-SY5Y cells and 53.87% in HMC3 cells compared to controls (Fig. 2G–I). We hypothesize that this dose-dependent ROS reduction is a consequence of the severe cytotoxic effects of AgNPs, which lead to a substantial decline in the overall cell population. A similar correlation was observed in our previous studies involving undifferentiated SH-SY5Y cells, human fibroblasts (BJ), and glioblastoma (U-87MG) cells, where high cytotoxicity was associated with decreased ROS levels (Skóra et al. 2022, 2023a, b). In summary, our findings demonstrate that internalization of small-size AgNPs in both neuronal and microglial models disrupts *redox* homeostasis, ultimately leading to cytotoxicity. Based on these results, as well as IC₅₀ parameters over the course of treatment, we selected 1 µg/mL AgNPs as a biologically active yet non-lethal dose, allowing further evaluation of small-size AgNP effects in the context of neuroinflammation and DAM-like phenotype induction. Moreover, a recent meta-analysis by Janzadeh et al., which reviewed 26 full research papers, demonstrated that AgNPs induce neurotoxicity in a dose-dependent manner at microgram-per-kilogram concentrations in animal models (Janzadeh et al. 2022). Similarly, Skalska et al. reported significant deposition of AgNPs in rat brain tissue at microgram concentrations, which was associated with synaptic dysfunction (Skalska et al. 2015).

### AgNPs affect the antioxidant system-related proteins as well as TLR4 expression in HMC3 cells

**Fig. 3:**
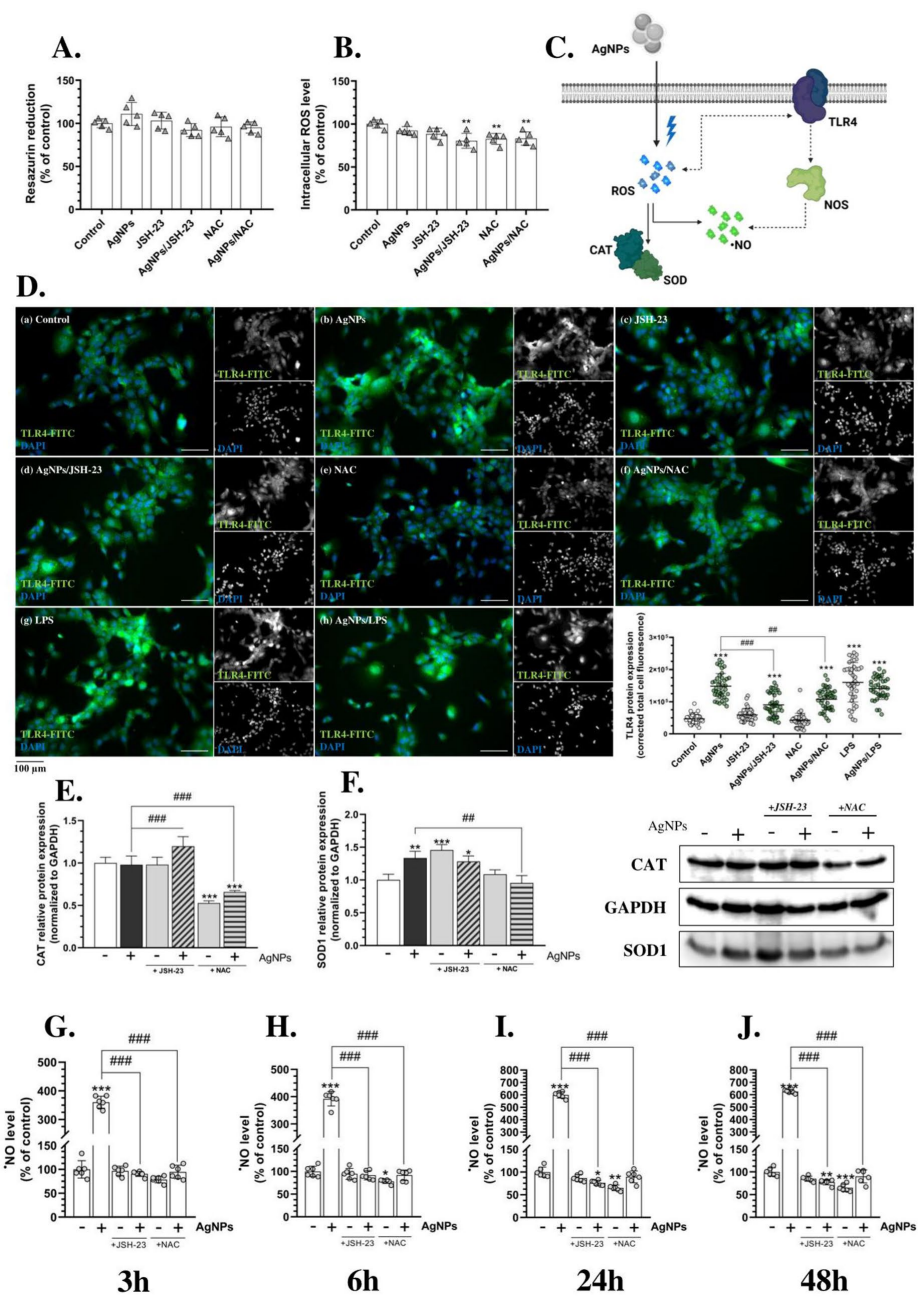
AgNP-induced impairment of ^•^NO homeostasis, coupled with TLR4 activation in microglial cells in vitro. Metabolic activity—24 h (**A**; *n* = 5), intracellular ROS levels—24 h (**B**; *n* = 5), IF staining of TLR4-FITC (green channel), DAPI (blue channel), and Corrected Total Cell Fluorescence measurements (**D**; *n* = 40); CAT (**E**) and SOD1 (**F**) protein expression—72 h (*n* = 3); as well as ^•^NO generation levels—3 h, 6 h, 24 h, and 48 h (**G**–**J**; *n* = 6) after treating the HMC3 cells with 1 µg/mL AgNPs, 10 µM JSH-23, or 4 mM NAC alone, or in co-treatment (AgNPs/JSH-23; AgNPs/NAC) for the indicated time points (transwell co-culture-based experiments). A schematic summary of the results is presented in the upper-right corner (**C**). LPS (1 µg/mL), alone or in co-treatment, was used as a positive control for IF staining. Imaging was performed at 200 × magnification, and the scale bar represents 100 µm. Data are presented as means ± SD. Statistically significant differences compared to the control are denoted as *, **, and *** for *p* < 0.05, *p* < 0.01, and *p* < 0.001, respectively (ANOVA, Tukey’s test). Statistically significant differences between specific groups are indicated as ## and ### for *p* < 0.01 and *p* < 0.001, respectively (*t*-test). The non-processed and edited images are available at 10.5281/zenodo.14756605. (color figure online)

Since ROS overproduction can lead to oxidative stress, which subsequently acts as a trigger factor for inflammation, we included two additional compounds in our experimental design: a nuclear factor kappa-light-chain-enhancer of activated B cells (NF-κB) selective inhibitor (JSH-23) as well as NAC, a potent antioxidant (Dodd et al. 2008; Rangaraju et al. 2018). A 24 h co-treatment of HMC3 cells in a monoculture with either AgNPs/JSH-23 or AgNPs/NAC did not result in significant differences in metabolic activity compared to AgNP-treated cells (Fig. 3A). However, after 24 h, intracellular ROS levels significantly decreased in NAC-treated cells as well as in AgNPs/NAC- and AgNPs/JSH-23-co-treated cells (Fig. 3B). These findings suggest that ROS induced by small-size AgNPs may contribute to oxidative stress, which in turn could serve as a primary pro-inflammatory factor in HMC3 cells.

In the next phase of our study, we established a transwell co-culture system of microglia (HMC3) and neurons (differentiated SH-SY5Y cells) to better simulate cell–cell interactions in AgNP-driven effects and facilitate future extrapolation of the results. To further investigate the hypothesis of AgNP-induced oxidative stress, we assessed the protein expression of two key antioxidant enzymes—catalase (CAT) and superoxide dismutase 1 (SOD1)—following long-term treatment (72 h) (Yilgor and Demir 2024). Our results revealed a significant increase in SOD1 protein expression by 33.57%, 45.51%, and 28.18% in HMC3 cells treated with AgNPs, JSH-23, and AgNPs/JSH-23, respectively, compared to the control (Fig. 3F). However, co-treatment with AgNPs and NAC abolished this effect (Fig. 3F). In contrast, CAT protein expression did not show significant changes in AgNPs-, JSH-23-, or AgNPs/JSH-23-treated cells. Notably, a substantial decrease in CAT expression was observed in NAC-treated (47.28% reduction) and AgNPs/NAC-co-treated cells (34.15% reduction) (Fig. 3E). These results confirm that small-size AgNPs induce oxidative stress in HMC3 cells co-cultured with a simplified neuron model, as demonstrated by the reversal of effects upon NAC co-treatment. Although no previous studies have conducted an in-depth analysis of AgNPs’ effects on microglia, particularly regarding CAT and SOD1 protein expression, numerous studies support the mechanism we propose. Fang et al. reported increased CAT, SOD, and glutathione peroxidase (GPx) activity in AgNP-treated hepatoma (HepG2) cells, while Vahabirad et al. observed oxidative stress-characteristic changes in CAT and SOD activity in the human breast cancer (SK-BR-3) cells (Fang et al. 2019; Vahabirad et al. 2024). Conversely, Docea et al. found that AgNPs exhibited antioxidant effects in male Wistar rats, as evidenced by total antioxidant capacity measurements (Docea et al. 2020). Moreover, Gonzalez-Carter et al. suggested that AgNPs have mitigating properties in LPS-stimulated N9 cells (Gonzalez-Carter et al. 2017). The discrepancies between these studies and our findings may be due to differences in the tested models, type of cells (liver, breast, or neuronal), or in nanoparticle size, which is significantly smaller in our study. Interestingly, the lack of significant changes in CAT activity in our study may be attributed to AgNPs’ ability to form an inhibitory complex with CAT, as first demonstrated by Liu et al. (Liu et al. 2020). Additionally, the observed increase in SOD1 protein expression following treatment or co-treatment with JSH-23 may be related to prolonged NF-κB inhibition (72 h), leading to secondary ROS generation as a result of cellular stress.

Given the reports, ROS can regulate specific inflammation-related proteins, such as Toll-like receptor 4 (TLR4), whose well-known external ligand is LPS (Kong et al. 2010). Deng et al. further demonstrated that TLR4 activation triggers the activity of inducible nitric oxide synthase (iNOS), an enzyme responsible for ^•^NO synthesis, resulting in elevated ^•^NO levels (Deng et al. 2015). Moreover, ROS can affect NOS activity in a dose-dependent manner as presented by Sun et al. (Sun et al. 2010). In addition to bacterial-derived LPS, several other TLR4 ligands have been identified as damage-associated molecular patterns (DAMPs) (Chen et al. 2009; Fang et al. 2011; Ma et al. 2017; Oseni et al. 2023). Our findings demonstrate a time-dependent increase in intracellular ^•^NO levels in HMC3 cells treated with 1 µg/mL AgNPs: 260.64% after 3 h, 292.29% after 6 h, 499.67% after 24 h, and 526.14% after 48 h, compared to the control (Fig. 3G–J). This effect correlates with ROS and NF-κB activity, as evidenced by the attenuation of AgNP effects following co-treatment with either JSH-23 or NAC (Fig. 3G–J). Importantly, enhanced TLR4 protein expression was observed in AgNP- and LPS-treated cells, as well as in AgNPs/JSH-23-, AgNPs/NAC-, and AgNPs/LPS-co-treated HMC3 cells (Fig. 3D). These findings suggest that AgNPs uptake induces oxidative stress, leading to increased antioxidant enzyme expression and TLR4 activation, ultimately resulting in inflammation and ^•^NO synthesis. However, this effect may also be influenced by cytokine and/or DAMP secretion, as neither NAC nor JSH-23 fully abolished AgNPs effects at the TLR4 level. Although we did not quantify specific cytokine secretion—which would have allowed us to make the above statement, Yang et al. reported increased pro-inflammatory interleukin-1 beta (IL-1β) levels in human blood monocytes in response to the AgNPs (Yang et al. 2012). Similarly, Li et al. demonstrated that AgNPs cause liver damage in mice, leading to mitochondrial release, which is considered a form of DAMP signaling (Li et al. 2021). Consequently, our subsequent analyses focused on ROS-, ^•^NO-, and potential TLR4-mediated downstream activation in 2D co-culture models, as well as their roles in DAM-like phenotypes and inflammation induced by treatment with small-size AgNPs.

### Oxidative stress-driven AgNPs-induced DAM-like phenotype in co-cultured microglia in vitro model

**Fig. 4:**
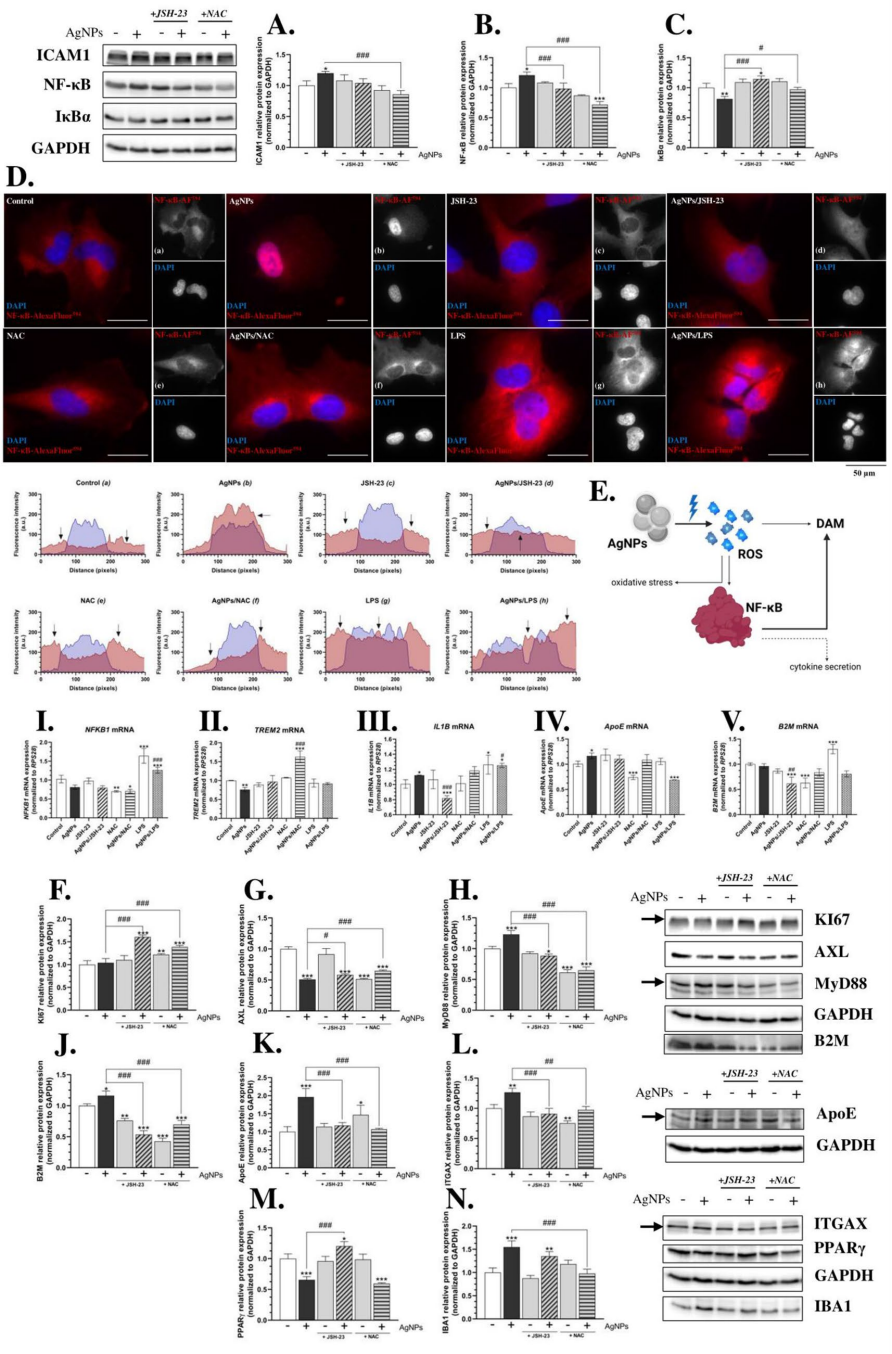
AgNPs trigger NF-κB nuclear translocation, followed by DAM-like phenotype induction and inflammation. Protein expression of ICAM1 (**A**), NF-κB (**B**), and IκBα (**C**)—72 h (*n* = 3); IF staining of NF-κB-AlexaFluor^594^ (red channel) and DAPI (blue channel)—90 min (**D**); intensity plots showing NF-κB-AlexaFluor^594^ nuclear translocation (a–h); mRNA expression of *NFKB1* (I), *TREM2* (II),* IL1B* (III), *ApoE* (IV), and *B2M* (V)−24 h (*n* = 3); and protein expression (*n* = 3) of KI67 (**F**), AXL (**G**), MyD88 (**H**), B2M (**J**), ApoE (**K**), ITGAX (**L**), PPARγ (**M**), and IBA1 (**N**) after treating HMC3 cells with 1 µg/mL AgNPs, 10 µM JSH-23, or 4 mM NAC alone, or in co-treatment (AgNPs/JSH-23; AgNPs/NAC) for the indicated time points (transwell co-culture-based experiments). A schematic summary of the results is presented at the right (**E**). LPS (1 µg/mL), alone or in co-treatment, was used as a positive control for IF staining and/or mRNA measurements. Imaging was performed at 630 × magnification, and the scale bar represents 50 µm. Data are presented as means ± SD. Statistically significant differences compared to the control are denoted as *, **, and *** for *p* < 0.05, *p* < 0.01, and *p* < 0.001, respectively (ANOVA, Tukey’s test). Statistically significant differences between specific groups are indicated as ## and ### for *p* < 0.01 and *p* < 0.001, respectively (*t*-test). The non-processed and colored images are available at 10.5281/zenodo.14884604. (color figure online)

The activation of NF-κB is tightly regulated by its translocation from the cytoplasm to the nucleus, where it controls the expression of inflammation-related genes (Tak and Firestein 2001; Badimon et al. 2020). Prolonged NF-κB activation, driven by sustained oxidative stress and inflammatory stimuli, may contribute to the induction of the DAM phenotype, reinforcing a neuroinflammatory state in microglia (Muzio et al. 2021). Our results showed that, just 90 min after treatment, AgNPs caused an increased fluorescence level of this protein in the nucleus of HMC3 cells (Fig. 4D, a). Interestingly, NAC prevented the nuclear translocation of NF-κB, indicating a primary role of ROS in the observed effect (Fig. 4D, e, f). This was further supported by our co-treatment experiments using JSH-23, which reduced nuclear fluorescence of NF-κB but did not entirely block its translocation after 90 min of treatment (Fig. 4D, c, d). Our further analysis focused on determining the expression of inflammation-related mRNAs regulated by NF-κB, particularly *NFKB1* and *IL1B*, to fully elucidate the mechanism of AgNPs action. After 24 h, *NFKB1* mRNA expression was not significantly altered in AgNP-treated microglial cells (Fig. 4I). However, a 12.13% increase in *IL1B* mRNA expression was observed, compared to the control (Fig. 4III). Notably, co-treatment with AgNPs/NAC reversed the effect of AgNPs, leading to a significant decrease in *NFKB1* gene expression by 29.04%, compared to the control (Fig. 4I). Similarly, co-treatment with either AgNPs/JSH-23 or AgNPs/NAC reversed or abolished the AgNP-induced increase in *IL1B* mRNA expression (Fig. 4III). Since *IL1B* transcription is regulated by NF-κB, these findings further support our hypothesis that AgNP-induced oxidative stress leads to NF-κB translocation and activation of specific genes, such as *IL1B*, a pro-inflammatory cytokine (Diep et al. 2022). Although our study is the first to demonstrate the pro-inflammatory properties of AgNPs in human microglial cells, the previous research has reported similar effects in in vivo models. For instance, Yousef et al. demonstrated that AgNPs (50 nm) induce neurotoxicity and neuroinflammation, accompanied by increased ^•^NO levels in the brains of male Wistar rats (Yousef et al. 2019). Additionally, Trickler et al. showed that AgNP-treated rats exhibit NF-κB pathway activation, leading to increased blood–brain barrier permeability (Trickler et al. 2010).

NF-κB activation is tightly regulated by IκBα, which inhibits NF-κB in its inactive state. Upon phosphorylation, IκBα undergoes degradation, allowing NF-κB to translocate to the nucleus via the canonical pathway (Hinz and Scheidereit 2014). Our results showed an 18.66% decrease in IκBα levels after treating HMC3 cells with AgNPs, an effect that was abolished by co-treatment with JSH-23 or NAC (Fig. 4B). Complementary results were observed in NF-κB protein expression, where AgNP treatment for 72 h led to a 20.79% increase in NF-κB protein levels compared to the control. This effect was reversed or abolished by co-treatment with JSH-23 or NAC (Fig. 4C). Additionally, we propose that AgNP-induced ROS activates the TLR4 receptor, which is supported by our findings on myeloid differentiation primary response 88 (MyD88) protein expression. MyD88 is responsible for transducing signals downstream of TLR4 (Ahmed et al. 2021). We observed a significant (23.01%, compared to the control) increase in MyD88 protein levels, while co-treatment the HMC3 with JSH-23 or NAC prevented these changes (Fig. 4H). Based on these findings, we suggest that oxidative stress induced by AgNPs activates both the canonical and receptor-dependent inflammatory pathways. However, we observed a chronic rather than acute activation, as these changes became evident after prolonged exposure (72 h). Importantly, the observed decrease in AXL protein expression (by 49.15%, compared to the control) and corresponding decrease in the peroxisome proliferator-activated receptor gamma (PPARγ) protein expression by 34.49%, compared to the control support the presence of the chronic inflammation after AgNPs-treatment in HMC3 cells (Weinger et al. 2011; Nerviani et al. 2024; Pearson et al. 2024) (Fig. 4G, M). This in turn is a hallmark of several neurodegenerative diseases, including AD and dementia (Kinney et al. 2018; Zhao et al. 2020).

NF-κB activation plays a crucial role in shaping the DAM phenotype, a specialized subtype of microglia linked to neurodegenerative disorders, like dementia and/or AD (Keren-Shaul et al. 2017; Deczkowska et al. 2018). Characterized by the upregulation of pro-inflammatory genes such as *IL1B*, along with DAM-associated markers like *ApoE*, *TREM2*, and *B2M*, these microglia exhibit sustained NF-κB signaling, driving chronic neuroinflammation and contributing to neurodegeneration (Krasemann et al. 2017; Butovsky and Weiner 2018). Our results showed that HMC3 cells treated for 72 h with small-size AgNPs exhibit a DAM-like increase in the expression of specific proteins, including intercellular adhesion molecule-1 (ICAM1) by 20.04%, β2 microglobulin (B2M) by 16.17%, apolipoprotein E (ApoE) by 96.09%, integrin subunit alpha X (ITGAX) by 26.59%, and calcium-binding adapter molecule 1 (IBA1, also known as allograft inflammatory factor-1, AIF-1), compared to the control (Fig. 4A, J–L, N). Importantly, in almost all tested proteins, co-treatment of the cells with either JSH-23 or NAC prevented these changes and/or reversed them (Fig. 4A, J–L, N). These observations are in line with the previous studies, demonstrating that microglial activation under pathological conditions involves similar molecular markers, particularly those associated with neuroinflammatory and phagocytic responses. Notably, they also emphasize the downregulation of *TREM2* in microglia (Keren-Shaul et al. 2017; Parhizkar et al. 2019). Although we did not analyze TREM2 protein expression, our experiments showed a similar decrease in its gene expression after 24 h of AgNP treatment in HMC3 cells (by 23.89% compared to the control) (Fig. 4II). In contrast, *ApoE* mRNA expression increased by 16.48% compared to the control following AgNP treatment, an effect that was reversed by co-treatment with AgNPs/NAC (Fig. 4IV). Meanwhile, *B2M* mRNA expression remained unchanged after AgNP exposure but decreased following co-treatment with AgNPs/NAC (Fig. 4V). These findings correlate with the observed protein expression patterns and align with the TREM2-dependent and subsequent TREM2-independent phases of DAM phenotype induction proposed by Friedman et al. (Friedman et al. 2018).

**Fig. 5:**
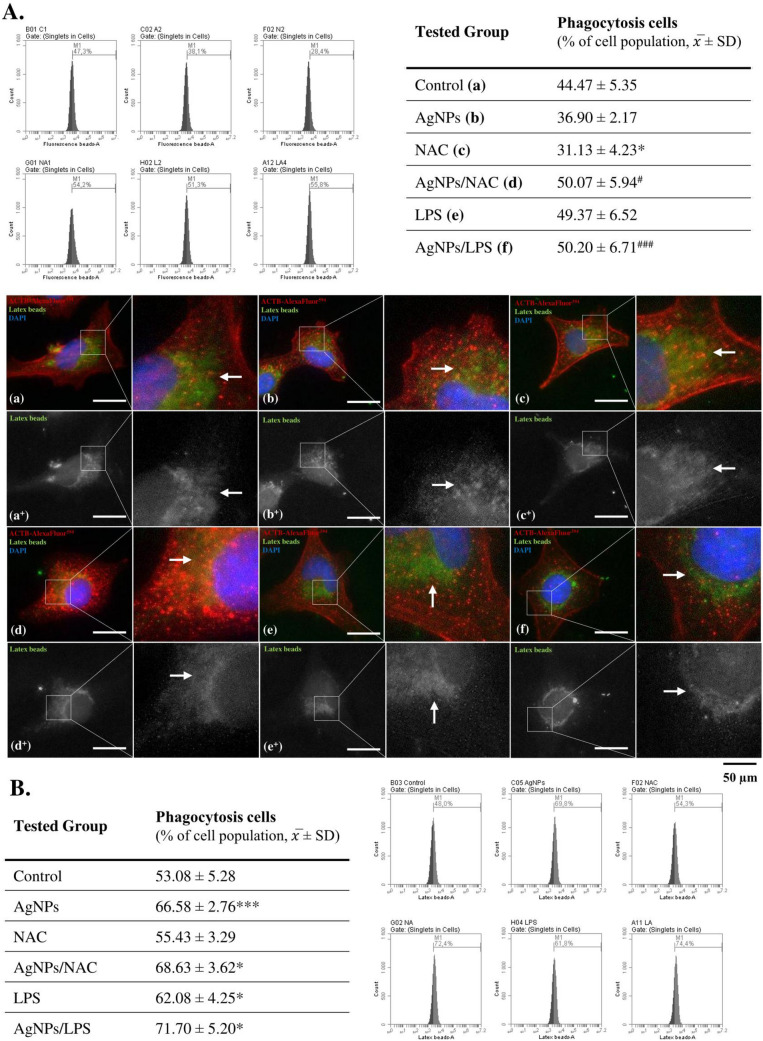
Small-size AgNPs affect the phagocytosis of human microglia cells in vitro. Phagocytosis assay results after treatment of HMC3 cells for 24 h (**A**) or 72 h (**B**) with 1 µg/mL AgNPs, 4 mM NAC, 1 µg/mL LPS, and in co-treatment (*n* = 3, mono-culture-based experiments). Representative graphs are shown next to the tables. The visualization of latex beads phagocytosis (green channel) by HMC3 cells exposed to the respective compounds is presented at the center of the figure. These cells were also immunostained with anti-ACTB-AlexaFluor^594^ antibodies (red channel) and DAPI (blue channel), with merged images provided. Imaging was performed at 630 × magnification. Data are presented as means ± SD. Statistically significant differences compared to the control are denoted as * and *** for *p* < 0.05 and *p* < 0.001, respectively (one-way ANOVA). Differences between AgNP-treated cells and AgNP co-treated cells are indicated as # and ### for *p* < 0.05 and *p* < 0.001, respectively (*t*-test). Images taken in the green channel (denoted as +) were processed using the Background Subtraction (radius: 300) ImageJ plug-in to enhance readability; however, non-processed and edited images are available at 10.5281/zenodo.14884638. (color figure online)

Given the evidence, the phagocytosis process in microglial cells plays a dual role in neurodegenerative diseases, contributing to both neuroprotection and neuroinflammation. Therefore, in the next part of our study, we decided to measure the phagocytic ability of HMC3 cells using fluorescent latex beads. Our results showed that after a short treatment period, AgNPs did not cause any significant changes in this parameter (Fig. 5A). Importantly, LPS-treated cells (positive control) also did not exhibit significant changes in phagocytic activity at this time point, suggesting that a short treatment period (i.e., 24 h) may not be sufficient to activate microglia. This contrasts with the findings of Dai et al., who reported that 6–24 h was sufficient to activate mouse microglial BV2 cells, as measured by cytokine release (Dai et al. 2015). Different origin of the cells may be a reason of the observed discrepancies. However, as proposed by Deczkowska et al., DAM-like cells are characterized by an upregulation of the phagocytosis-related genes expression, which is similar to the increased corresponding proteins expressions presented in our study after long-time of exposure (Deczkowska et al. 2018). Therefore, we extended our study by measuring the phagocytic ability of HMC3 cells after 72 h. The results supported the DAM-related mechanism proposed in the cited study, as AgNPs significantly increased the phagocytic ability of HMC3 cells by 13.50% compared to the control (Fig. 5B). Notably, NAC was unable to prevent these changes at this time point, which may be a result of too high oxidative stress in a such long-term exposition (Fig. 5B). This is further justified by studies from Kim and Choi, as well as Dalzon et al., which demonstrate alterations in phagocytosis-related genes and inflammatory responses in macrophages (Kim and Choi 2012; Dalzon et al. 2019). Finally, we demonstrated the upregulation of ITGAX and ICAM1 protein expression, whose role in phagocytosis is indisputable (also linked with DAM). This is further supported by Zhong et al., who identified—also postulated by us—an ROS-induced intracellular axis involving ROS–TLR4–ICAM1–phagocytosis (Zhong et al. 2021). Taking above into account, we propose that AgNPs activate a DAM-like phagocytic protein pattern as a result of oxidative stress, inflammation, and probably external-related TLR4 activation.

### AgNPs-induced DAM-like phenotype affects the function of the neurons in co-cultured model in vitro

**Fig. 6:**
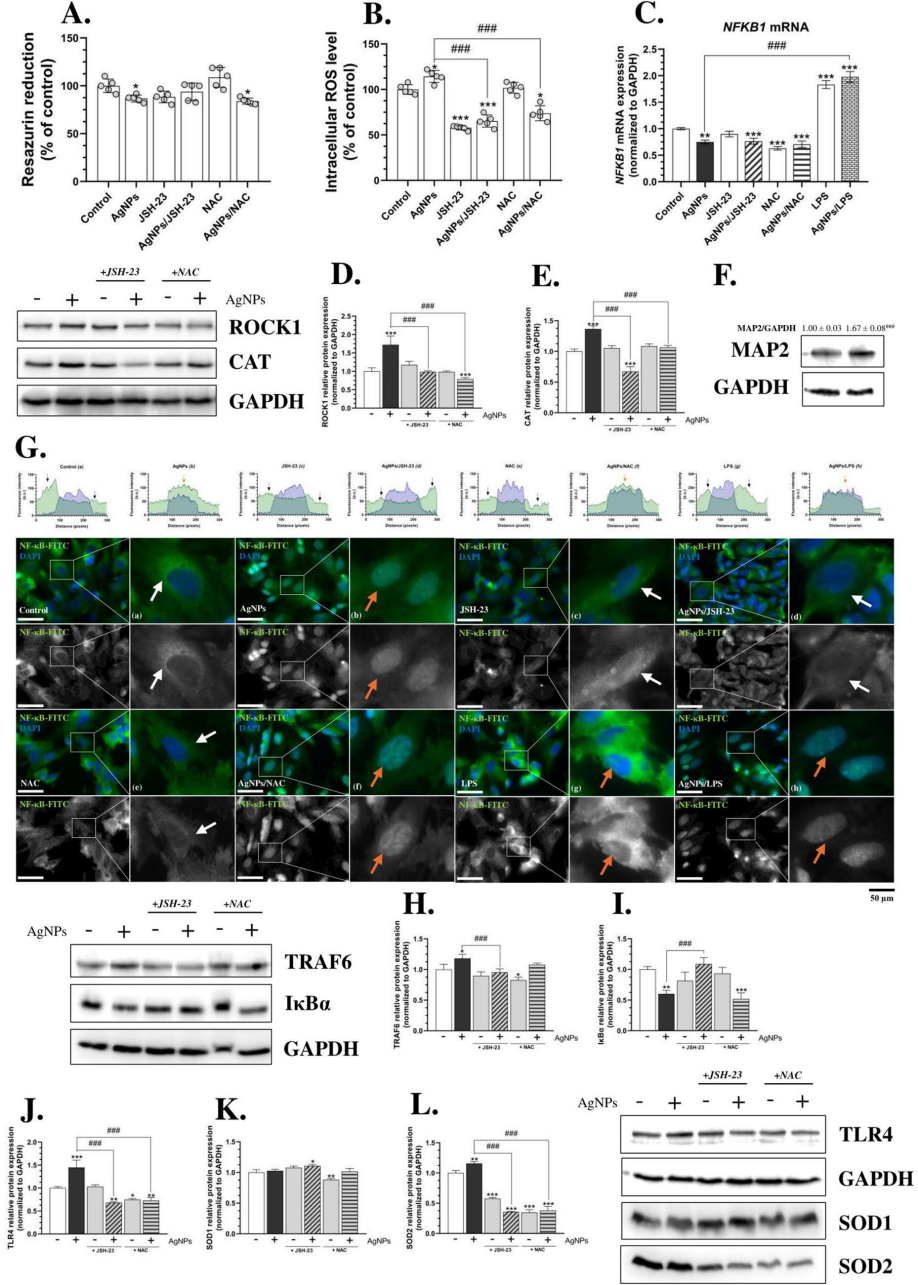
Small-size AgNPs-induced oxidative stress-driven inflammation in co-cultured, differentiated SH-SY5Y cells. The metabolic activity—72 h (**A**; *n* = 5, mono-culture-based experiments), intracellular ROS level—72 h (**B**; *n* = 5; mono-culture-based experiments), *NFKB1* mRNA expression—24 h (**C**; *n* = 3; transwell co-culture-based experiments), the protein expression (n = 3, transwell co-culture-based experiments) of ROCK1 (**D**), CAT (**E**), IF staining of NF-κB-FITC (green channel) and DAPI (blue channel)—24 h (**G**); intensity plots showing NF-κB-FITC nuclear translocation (a – h), the protein expression (n = 3; transwell co-culture-based experiments) of TRAF6 (**H**), IκBα (**I**), TLR4 (**J**), SOD1 (**K**), and SOD2 (**L**)—72 h after treating differentiated SH-SY5Y cells with 1 µg/mL AgNPs, 10 µM JSH-23, or 4 mM NAC alone, or in co-treatment (AgNPs/JSH-23; AgNPs/NAC) for the indicated time points. For IF experiments, the CM from HMC3 was used. The expression of MAP2 at 0th and 7th day of differentiation is shown to prove the efficiency of the protocol (**F**; *n* = 3). The LPS (1 µg/mL), alone or in co-treatment, was used as a positive control for IF staining. The orange arrows indicate the obvious NF-κB translocation. Imaging was performed at 630 × magnification, and the scale bar represents 50 µm. Data are presented as means ± SD. Statistically significant differences compared to the control are denoted as *, **, and *** for *p* < 0.05, *p* < 0.01, and *p* < 0.001, respectively (ANOVA, Tukey’s test). Statistically significant differences between specific groups are indicated as ## and ### for *p* < 0.01 and *p* < 0.001, respectively (*t*-test). The non-processed and colored images are available at 10.5281/zenodo.14884681. (color figure online)

**Fig. 7:**
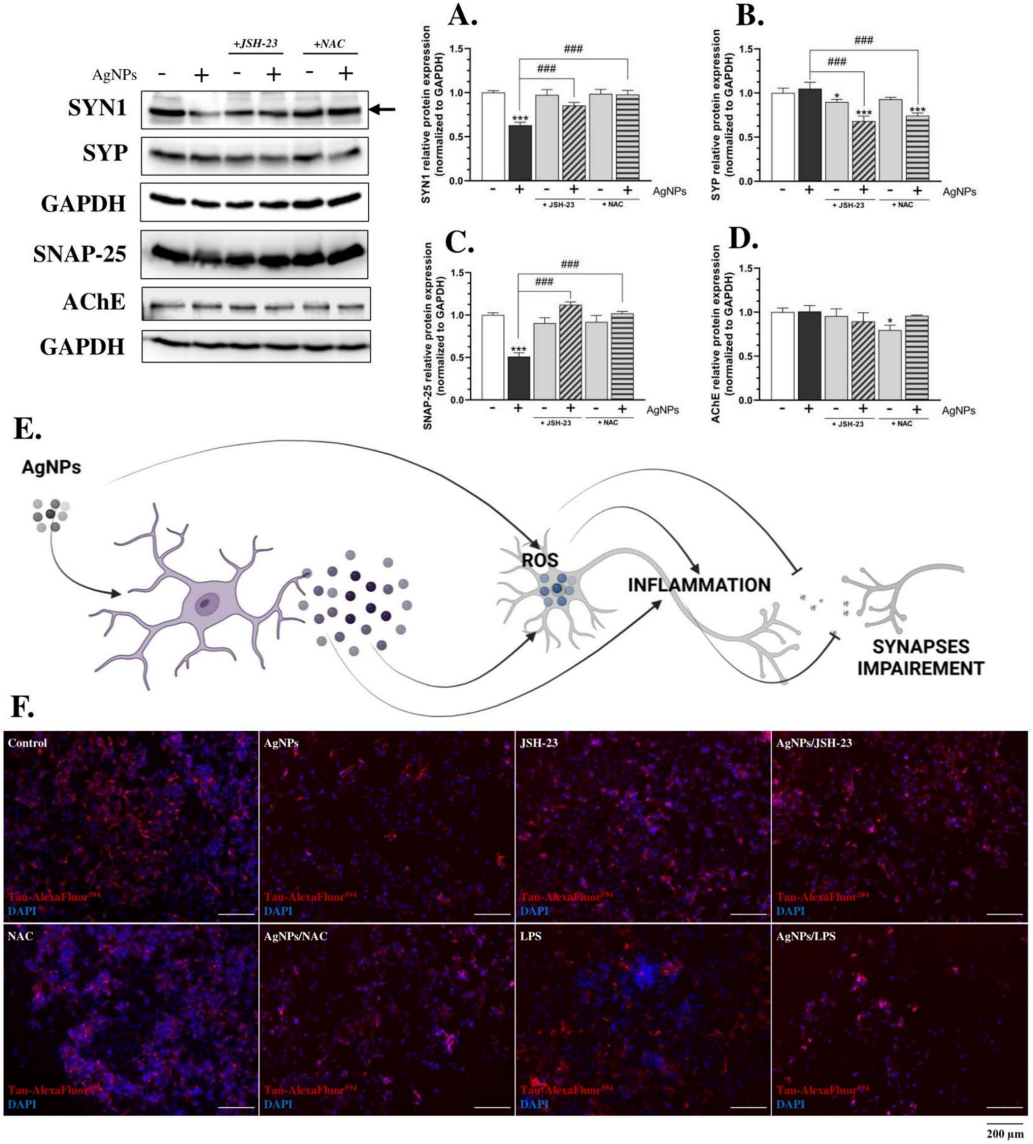
Synapses dysfunction in differentiated SH-SY5Y cells (neuron model) after exposure of AgNPs in co-culture conditions. The protein expression (n = 3; transwell co-culture-based experiments) of SYN1 (**A**), SYP (**B**), SNAP-25 (**C**), and AChE (**D**)—72 h, IF staining of Tau-AlexaFluor^594^ (red channel) and DAPI (blue channel)—24 h (**F**) after treating differentiated SH-SY5Y cells with CM, containing 1 µg/mL AgNPs, 10 µM JSH-23, or 4 mM NAC alone, or in co-treatment (AgNPs/JSH-23; AgNPs/NAC) for the indicated time points. The scheme, summarizing the obtained results is presented at the center (**E**). The LPS (1 µg/mL), alone or in co-treatment, was used as a positive control for IF staining. Imaging was performed at 100 × magnification, and the scale bar represents 200 µm. Data are presented as means ± SD. Statistically significant differences compared to the control are denoted as *, and *** for *p* < 0.05, and *p* < 0.001, respectively (ANOVA, Tukey’s test). Statistically significant differences between specific groups are indicated as ### for *p* < 0.001 (*t*-test). The non-processed and colored images are available at 10.5281/zenodo.14884657. (color figure online)

In this part of the study, we repeated the experiment with co-treatment using JSH-23 and NAC in a mono-culture of chosen neuron model, i.e., differentiated SH-SY5Y cells. The observed decrease in metabolic activity after 24 h of treatment was similar to that in the dose-dependent experiments (Fig. 6A). While NAC co-treatment did not prevent AgNP-induced cytotoxicity, JSH-23 induced reversed effect. This aligns with the intracellular ROS levels, where both JSH-23 and NAC reversed the effects of AgNPs, though JSH-23 had a stronger impact. Based on this, we believe that the mechanism of AgNP action in neurons resembles that in microglia, involving increased ROS, oxidative stress, and inflammation. Similar cytotoxic effects of AgNPs were previously reported by Park et al. in undifferentiated SH-SY5Y cells and by Weldon et al. in mouse organotypic brain cultures (Park et al. 2017; Weldon et al. 2018). Next, we have performed the IF staining of Tau protein to confirm the above-described dependency as this protein is characteristic for neuron cytoskeleton and may be used as an indicator of overall cell condition (Brandt et al. 2020). The obtained results show decrease number of neurons after treatment with AgNPs (visualized as lower level of red fluorescence), while this effect was reversed in AgNPs/JSH-23 and AgNPs/NAC co-treated cells (Fig. 7F).

Our further investigations focused on AgNP-induced DAM HMC3 cells’ effects on neurons using the transwell co-culture model. Our results confirm the oxidative stress-dependent action of AgNPs in neurons, as evidenced by increased CAT and SOD2 protein expression by 36.60% and 15.87%, respectively, after 72 h of AgNP treatment (Fig. 6E, L). Notably, in AgNP/JSH-23- and AgNP/NAC-co-treated cells, this effect was reversed, supporting our hypothesis and aligning with findings in microglia cells (Fig. 6E, L). Additionally, Dąbrowska-Bouta et al. and Dziendzikowska et al. reported similar pro-oxidative tendencies in rats exposed to 10 nm citrate-coated AgNPs (Dąbrowska-Bouta et al. 2019; Dziendzikowska et al. 2022).

Considering previous studies, AgNP-induced oxidative stress and inflammation contribute to DAM in HMC3 cells, characterized by increased *IL1B* mRNA expression, a key pro-inflammatory cytokine (Ren and Torres 2009). We propose that neuronal inflammation is triggered both intracellularly by AgNP-induced ROS via the IκBα/NF-κB pathway and may be also induced extracellularly by cytokines and/or DAMPs secreted by HMC3 cells. This is supported by NF-κB co-localization results, which showed nuclear accumulation of NF-κB fluorescence in AgNP-treated neurons after 24 h (Fig. 6G, b). While JSH-23 (an NF-κB inhibitor) mitigated this effect, AgNPs/LPS-co-treatment resulted in a summative effect, further promoting protein translocation. NAC was unable to fully block AgNP-induced translocation but did mitigate the effect (Fig. 6G, d, f, h). Although a 24.94% decrease in *NFKB1* mRNA expression was observed within this timeframe, we believe this discrepancy may be due to a positive feedback loop (Fig. 6C). In JSH-23-, AgNPs/JSH-23-, NAC-, and AgNPs/NAC-treated cells, *NFKB1* gene expression decreased by 23.61%, 37.01%, and 29.41%, respectively, likely due to the mitigating properties of these compounds (Fig. 6C). Kumar et al. similarly demonstrated that JSH-23 prevents NF-κB-dependent oxidative stress and inflammation (including NF-κB translocation) in Sprague Dawley rat sciatic nerves (Kumar et al. 2011). Likewise, Kim et al. and Shah et al. showed that AgNP-induced ROS effects were prevented in HepG2 and 16HBE cells, respectively (Kim et al. 2009; Shah et al. 2020). The observed 39.79% decrease in IκBα protein expression in AgNP-treated neurons, and its reversal after JSH-23 co-treatment, further supports inflammation induction (Fig. 6I).

**Fig. 8:**
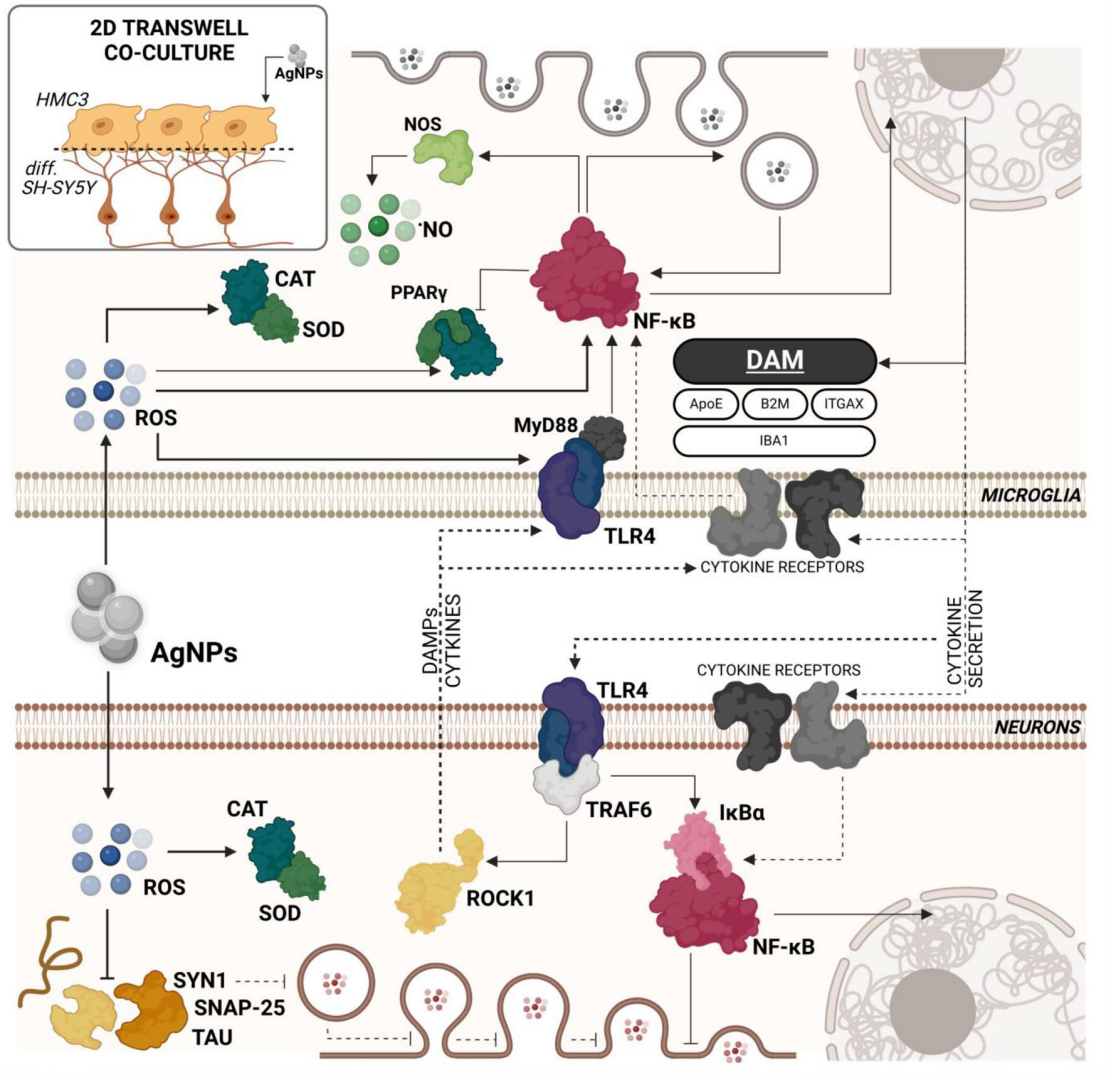
Proposed mechanism of DAM-like phenotype and neurodegeneration induction in co-cultured microglia/neurons in vitro. The dotted lines represent probably interactions

Additionally, increased TLR4 expression suggests an extracellular inflammation mechanism, similar to that observed in HMC3 cells. This is justified by the 44.76% increase in TLR4 expression in AgNP-treated neurons (Fig. 6J). These cells also exhibited an 18.11% increase in TRAF6 protein expression, a key intracellular signal transducer of TLR4 activation (Fig. 6H) (Walsh et al. 2015). Notably, TRAF6 also mediates pro-inflammatory signaling via IL-1βR1, IL-17R, and TNFRs (Wang et al. 2020). Although we did not measure cytokine and/or DAMP secretion, the increased *IL1B* mRNA expression in HMC3 cells suggests that AgNP-induced DAM in microglia triggers neuronal inflammation. TLR4 activation may also result from neuron-derived DAMPs in response to oxidative stress, a hallmark of many neurodegenerative diseases (Zhou et al. 2020). Additionally, AgNP-treated neurons showed a 72.24% increase in ROCK1 protein expression (Fig. 6D). Although in primary microglia, however, Glotfelty et al. evidenced the presence of TLR4-ROCK1 intracellular axis, which is activated as a response to the TLR4-induced inflammation and indicated its role in the cytoskeleton stabilization (Glotfelty et al. 2023). Importantly, in most cases, JSH-23 and NAC co-treatment abolished or significantly reduced AgNP-induced effects. Based on our findings and previous studies, we demonstrate that AgNPs activate a DAM-like phenotype in human microglia, leading to neuronal inflammation via both extracellular and intracellular stimuli. However, we are aware that the chosen microglial model (HMC3 cells) has specific limitations, and the obtained results should be validated using more physiologically relevant models, such as human induced pluripotent stem cell-derived microglia, as well as in vivo experiments (Dello Russo et al. 2018) (Fig. 8).

The final part of our study aimed to determine the effect of AgNP-induced disruptions on specific neuronal proteins involved in synapse-related transport and intercellular brain communication, including Synoviolin I (SYN1), Synaptophysin (SYP), synaptosome-associated protein-25 (SNAP-25), and acetylcholinesterase (AChE) (Yagishita et al. 2008; Luo et al. 2018; Marucci et al. 2021; Liu et al. 2022). Our results indicate that AgNP-induced ROS and/or inflammation may impair intercellular communication. Specifically, we observed a 37.09% decrease in SYN1 protein expression in AgNP-treated neurons compared to the control (Fig. 7A). Additionally, SNAP-25 protein levels were reduced by 48.94% in the same conditions (Fig. 7C). Notably, co-treatment with either JSH-23 or NAC effectively prevented these AgNP-induced reductions in both proteins (Fig. 7A, C). Previous studies have shown that downregulation of *SYN1* and *SNAP-25* gene expression may be associated with the early stages of AD and PD (Wang et al. 2023). Furthermore, recent findings by Cai et al. demonstrated reduced intercellular communication—measured by extracellular vesicle levels—in AD patients, alongside decreased *Syn1* and *Snap-25* mRNA expression in an AD mouse model (Cai et al. 2024). Although we have not detected any significant changes of the AChE as well as SYP proteins expression after treatment of AgNPs, the literature data in this question are inconsistent. Skalska et al. demonstrated that oral exposure to AgNPs in rats leads to a reduction in Syp levels, indicating synaptic damage, whereas Chang et al. reported that AgNPs induce synaptic transport loss by triggering the overexpression of Syp in male ICR mice (Skalska et al. 2015; Chang et al. 2022). This discrepancy suggests that the effects of AgNPs on synaptic proteins may be cell-specific and require further investigation. Nevertheless, the findings discussed above suggest that AgNPs induce synaptic disruption, which may have pathological relevance in neurodegenerative diseases.

## Conclusions

In this study, we report that small-size AgNPs can induce a DAM-like phenotype in a simplified model of human microglial cells through activation of the NF-κB pathway, driven by oxidative stress, as well as extracellular activation of specific receptors, such as TLRs. An increased level of ^•^NO was also detected. The observed phenotype may be probably related to the secretion of cytokines, which interact with specific cytokine-related receptors in neuronal cells, ultimately triggering extracellularly-induced inflammation. Furthermore, the uptake of AgNPs by these cells induces excessive ROS generation and the activation of antioxidant proteins, such as CAT and SOD. In cases of insufficiency, this response may further amplify inflammation. This process impairs synapse-dependent signaling pathways in tested neurons cells, as indicated by the altered expression of SNAP-25 and SYN1 proteins. Although this study was conducted using in vitro models, we believe our findings provide a strong foundation for further in vivo research on the safety of small-size AgNPs in humans.

## Supplementary Information

Below is the link to the electronic supplementary material.

Supplementary file1 (PPTX 25250 KB)

## Data Availability

Data are available on reasonable request and/or are stored in open repository (Zenodo).
