# Supplementary material for: Oxidative stress-driven disease-associated microglia (DAM)-like polarization in human microglia (HMC3) cells exposed to small-size silver nanoparticles in a transwell co-culture system with neurons (cholinergic differentiated SH-SY5Y) cells in vitro
Source: Arch Toxicol. 2025 Sep 17;100(1):207–29. doi: 10.1007/s00204-025-04183-0 (PMC12858520; doi:10.1007/s00204-025-04183-0)
Supplement: Supplementary file 1 — Supplementary file1 (PPTX 25250 KB) [file 204_2025_4183_MOESM1_ESM.pptx]

## Slide 1
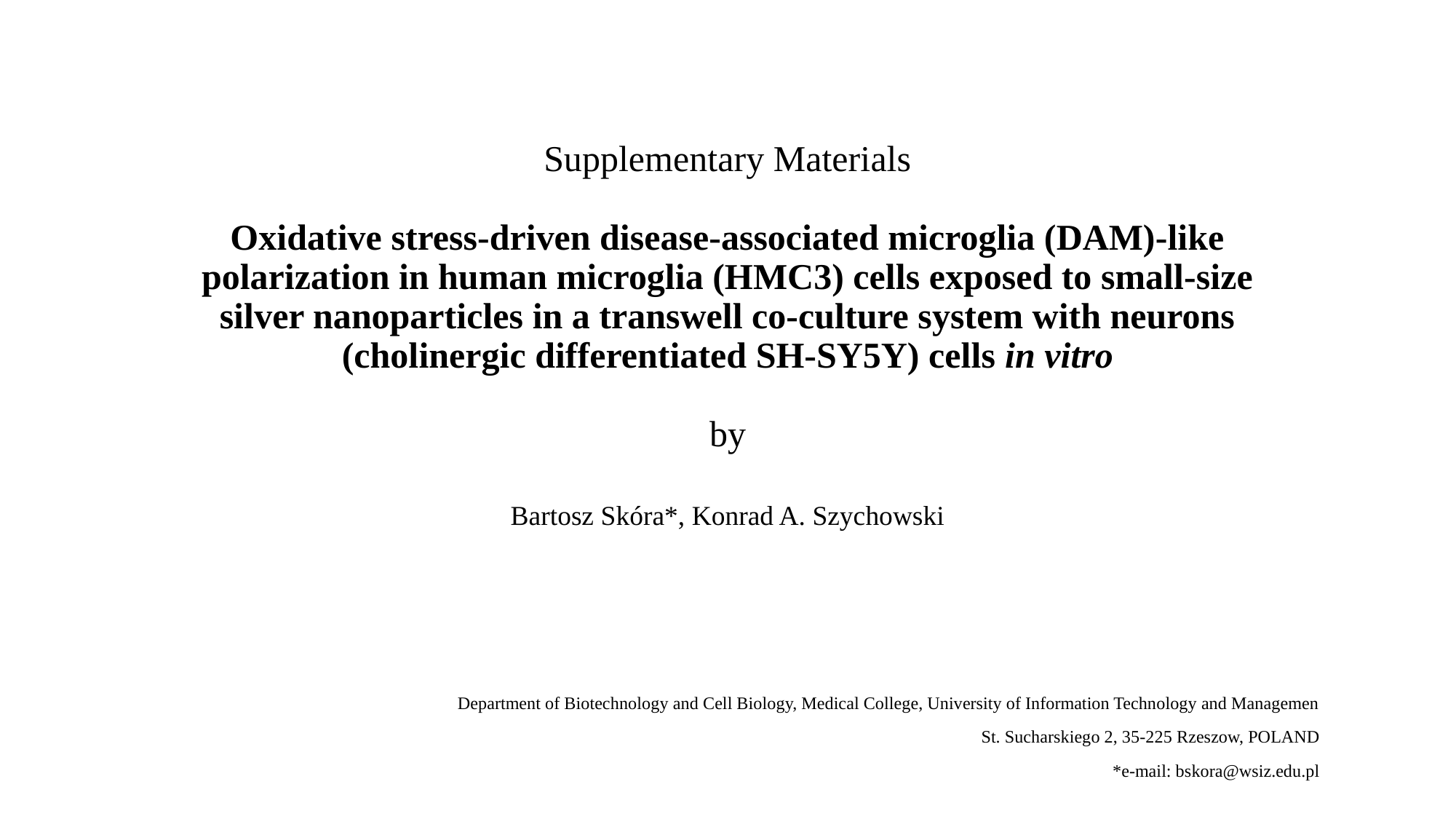

# Supplementary Materials Oxidative stress-driven disease-associated microglia (DAM)-like polarization in human microglia (HMC3) cells exposed to small-size silver nanoparticles in a transwell co-culture system with neurons (cholinergic differentiated SH-SY5Y) cells in vitro by
Bartosz Skóra*, Konrad A. Szychowski
Department of Biotechnology and Cell Biology, Medical College, University of Information Technology and Managemen
St. Sucharskiego 2, 35-225 Rzeszow, POLAND
*e-mail: bskora@wsiz.edu.pl

## Slide 2
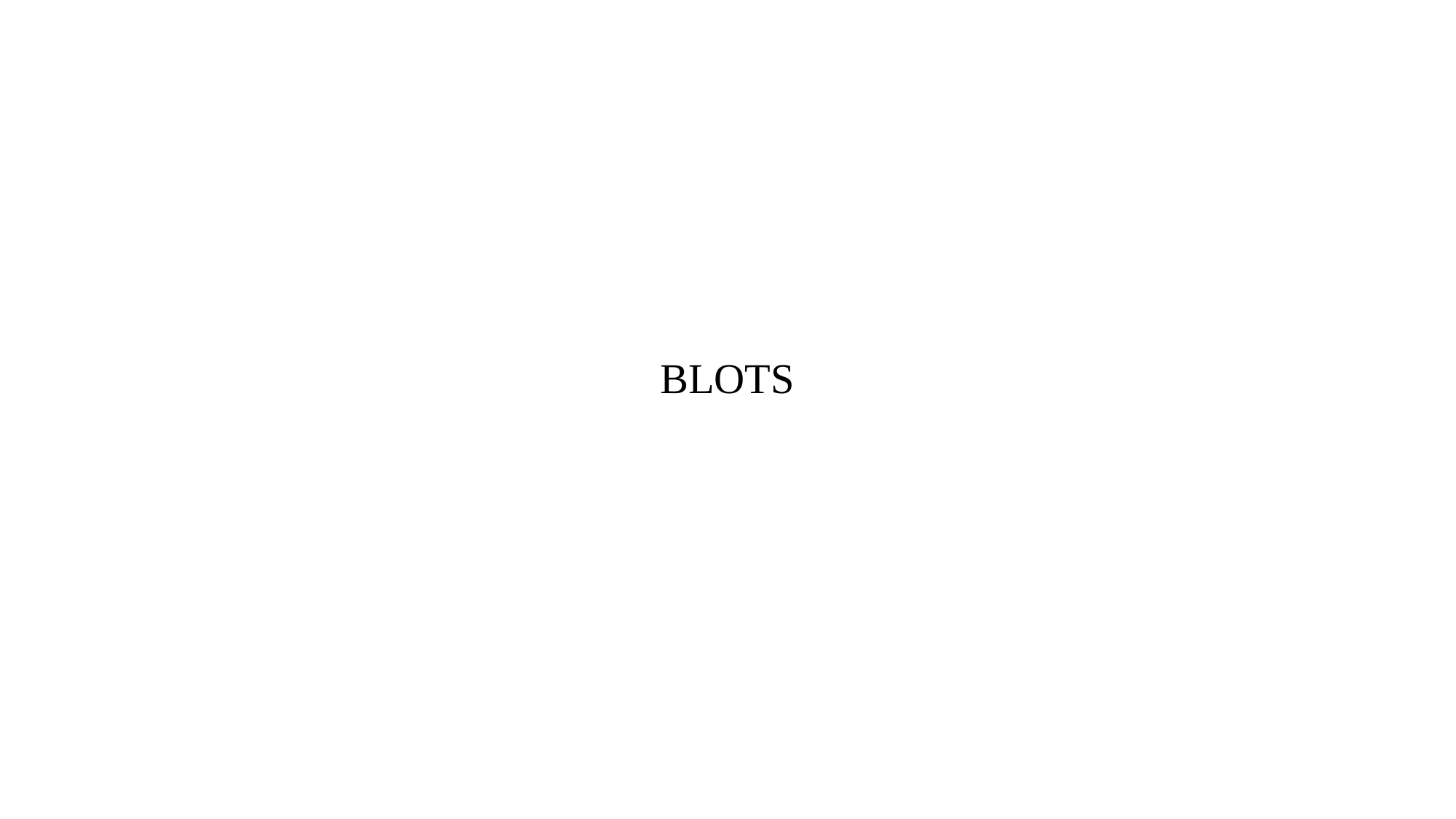

BLOTS

## Slide 3
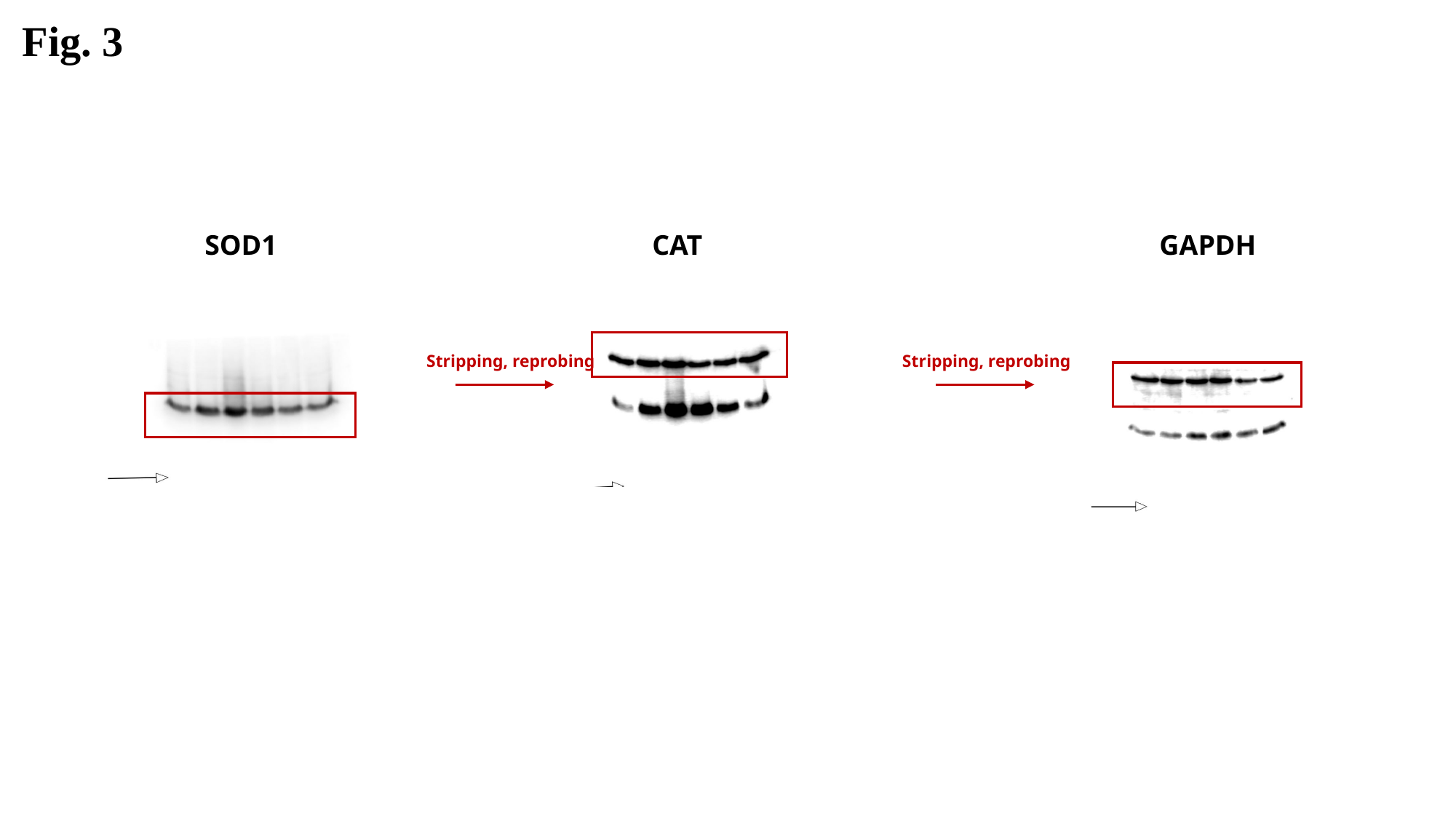

Fig. 3
SOD1
CAT
GAPDH
Stripping, reprobing
Stripping, reprobing

## Slide 4
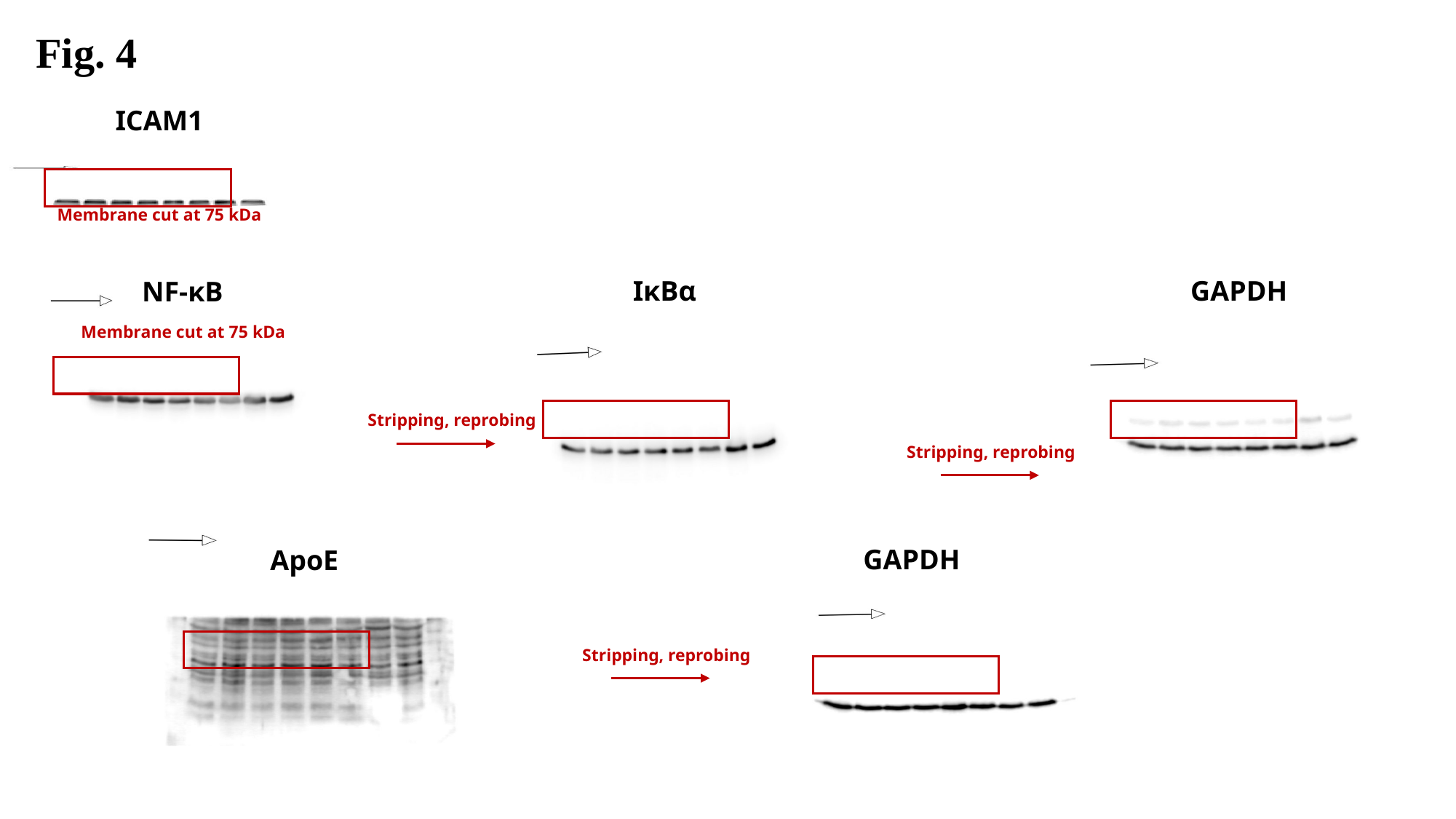

Fig. 4
ICAM1
Membrane cut at 75 kDa
IκBα
GAPDH
NF-κB
Membrane cut at 75 kDa
Stripping, reprobing
Stripping, reprobing
GAPDH
ApoE
Stripping, reprobing

## Slide 5
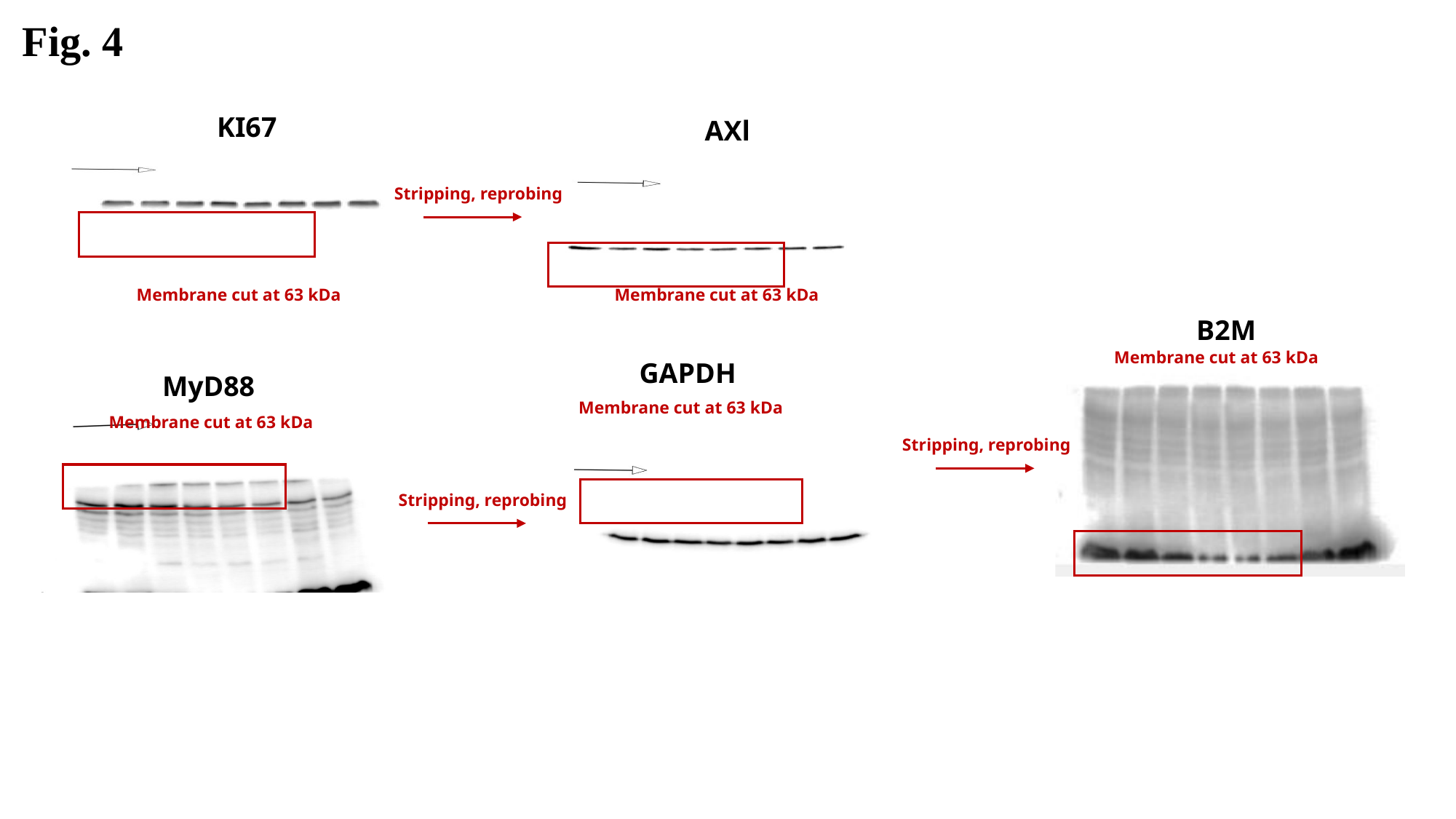

Fig. 4
KI67
AXl
Stripping, reprobing
Membrane cut at 63 kDa
Membrane cut at 63 kDa
B2M
Membrane cut at 63 kDa
GAPDH
MyD88
Membrane cut at 63 kDa
Membrane cut at 63 kDa
Stripping, reprobing
Stripping, reprobing

## Slide 6
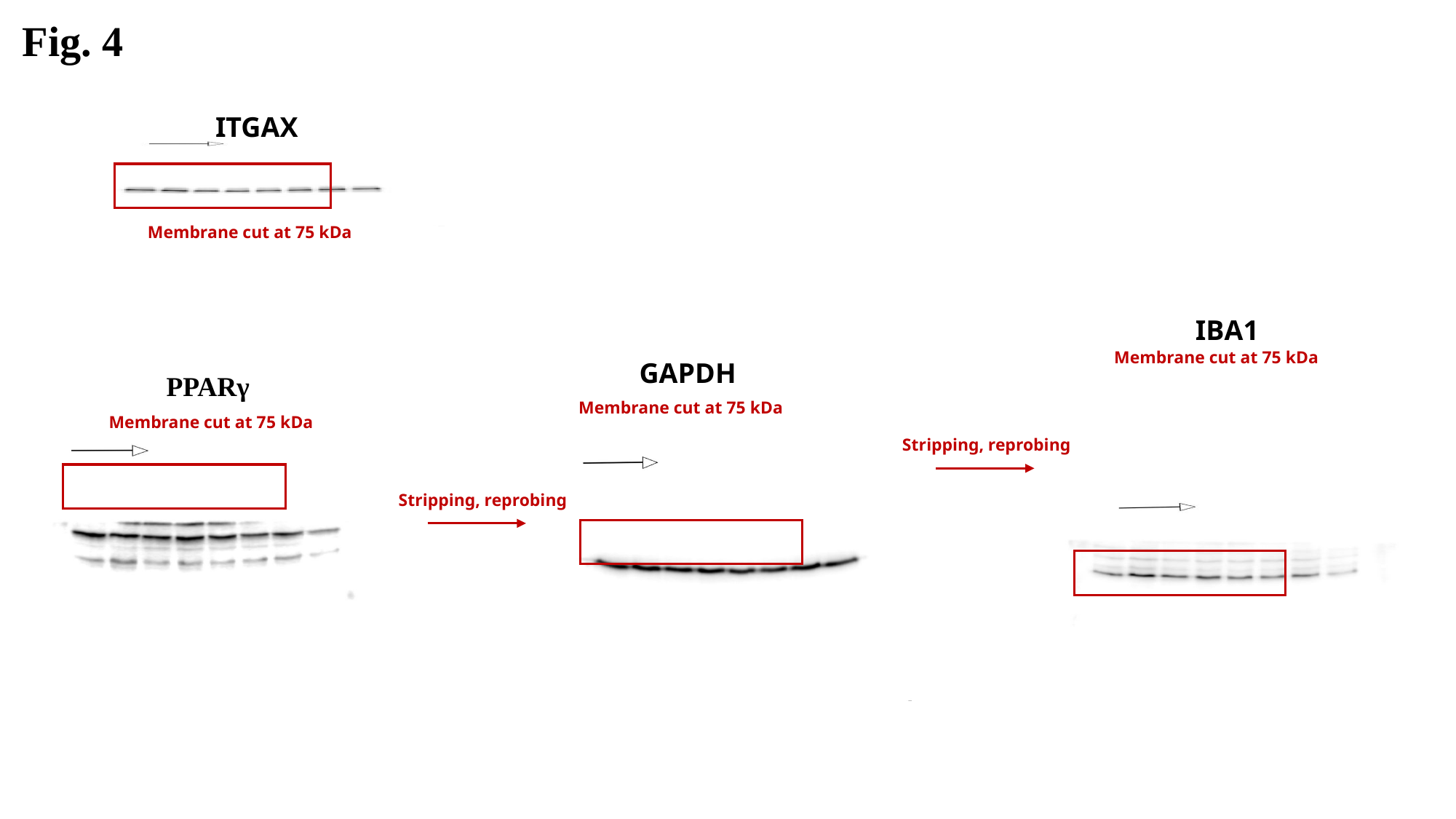

Fig. 4
ITGAX
Membrane cut at 75 kDa
IBA1
Membrane cut at 75 kDa
GAPDH
PPARγ
Membrane cut at 75 kDa
Membrane cut at 75 kDa
Stripping, reprobing
Stripping, reprobing

## Slide 7
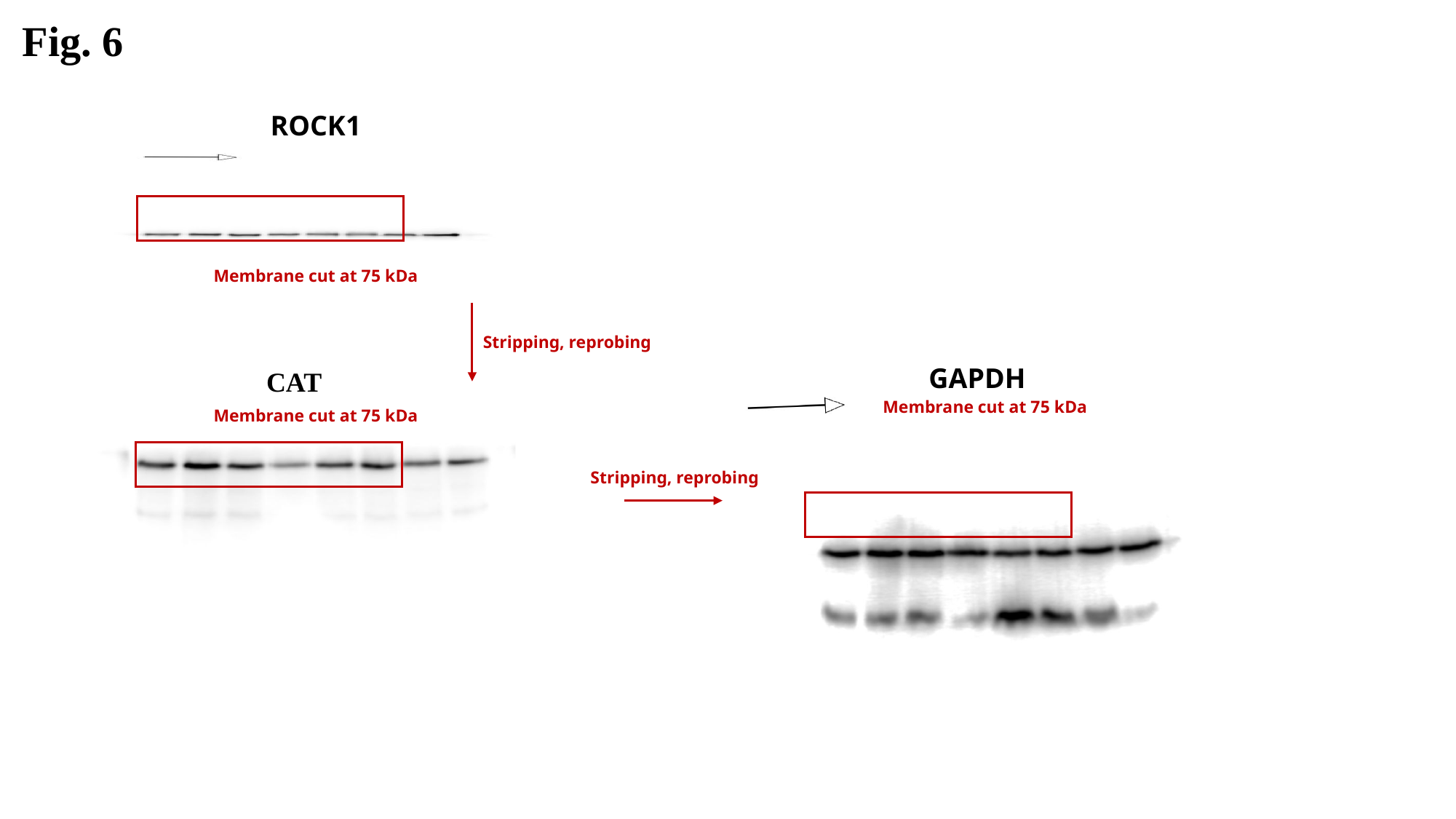

Fig. 6
ROCK1
Membrane cut at 75 kDa
Stripping, reprobing
GAPDH
CAT
Membrane cut at 75 kDa
Membrane cut at 75 kDa
Stripping, reprobing

## Slide 8
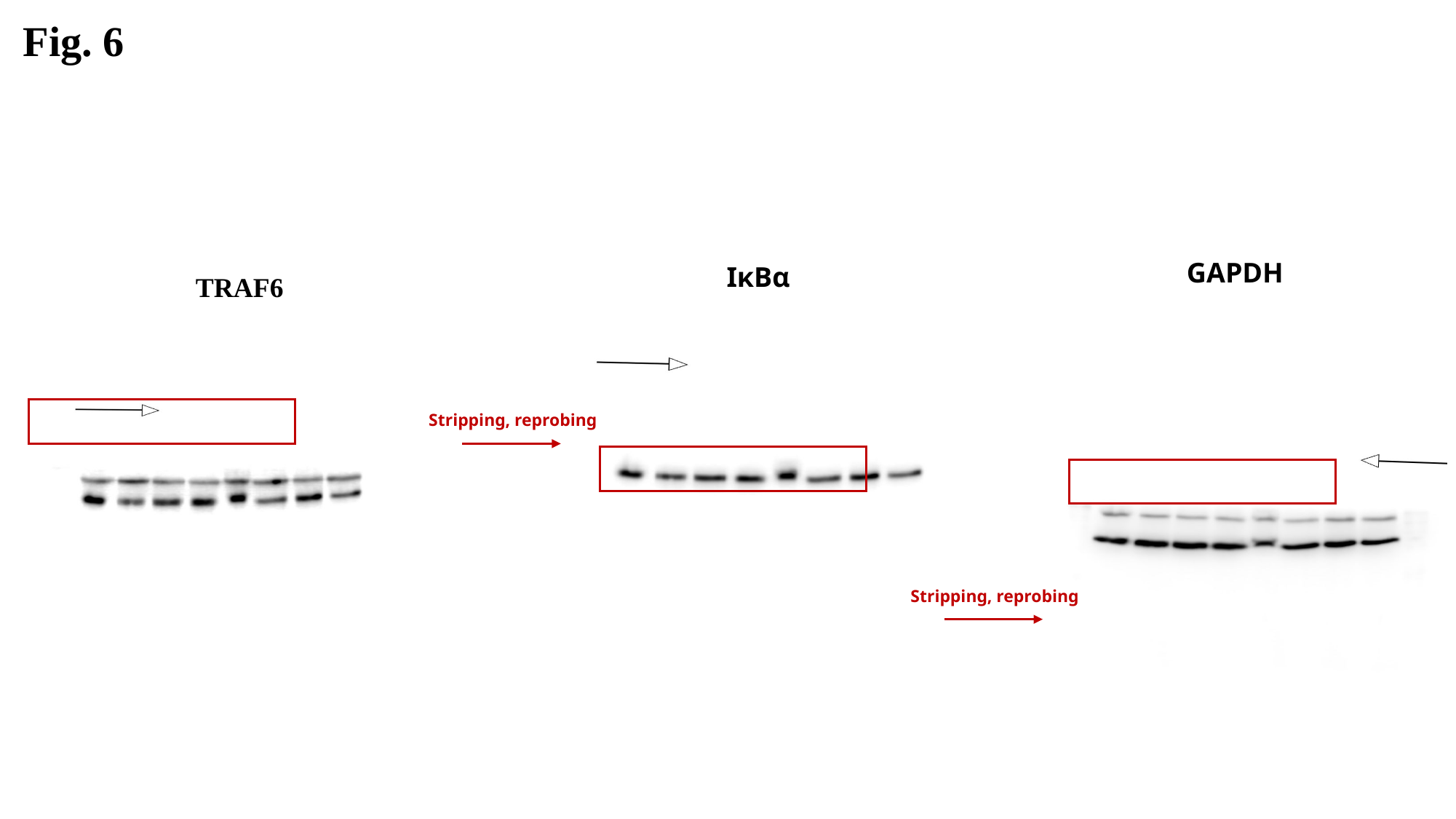

Fig. 6
GAPDH
IκBα
TRAF6
Stripping, reprobing
Stripping, reprobing

## Slide 9
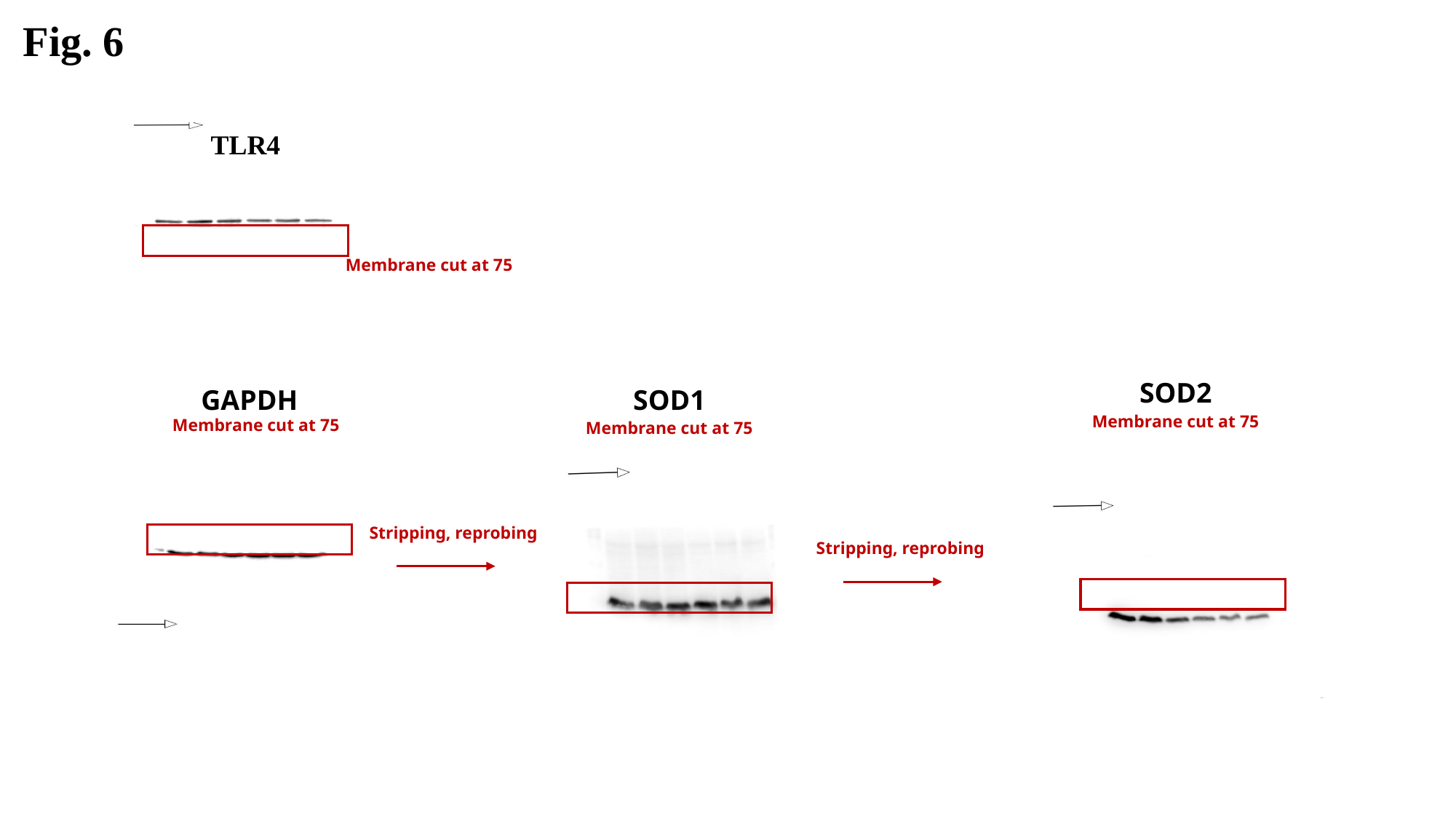

Fig. 6
TLR4
Membrane cut at 75
SOD2
GAPDH
SOD1
Membrane cut at 75
Membrane cut at 75
Membrane cut at 75
Stripping, reprobing
Stripping, reprobing

## Slide 10
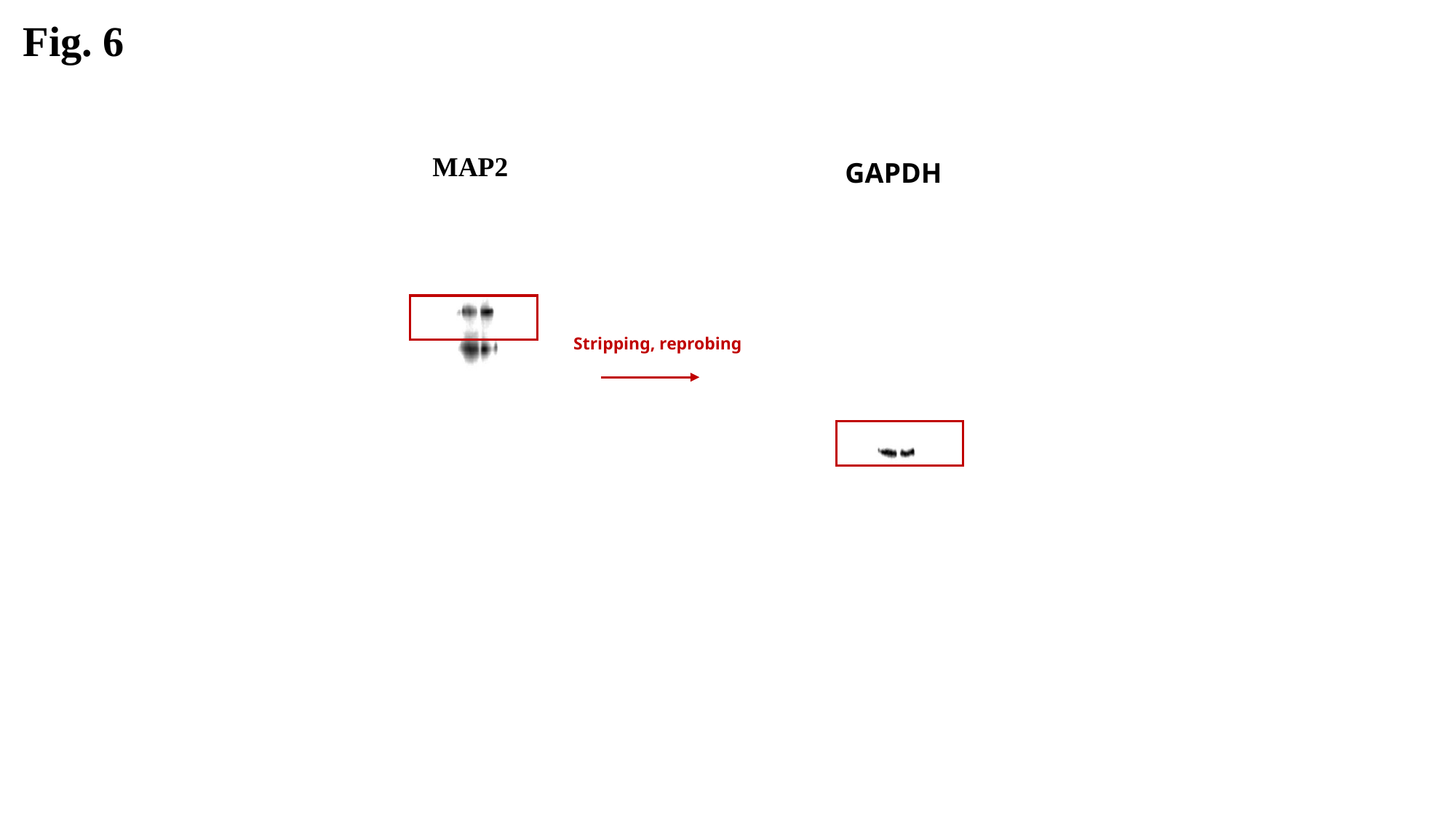

Fig. 6
MAP2
GAPDH
Stripping, reprobing

## Slide 11
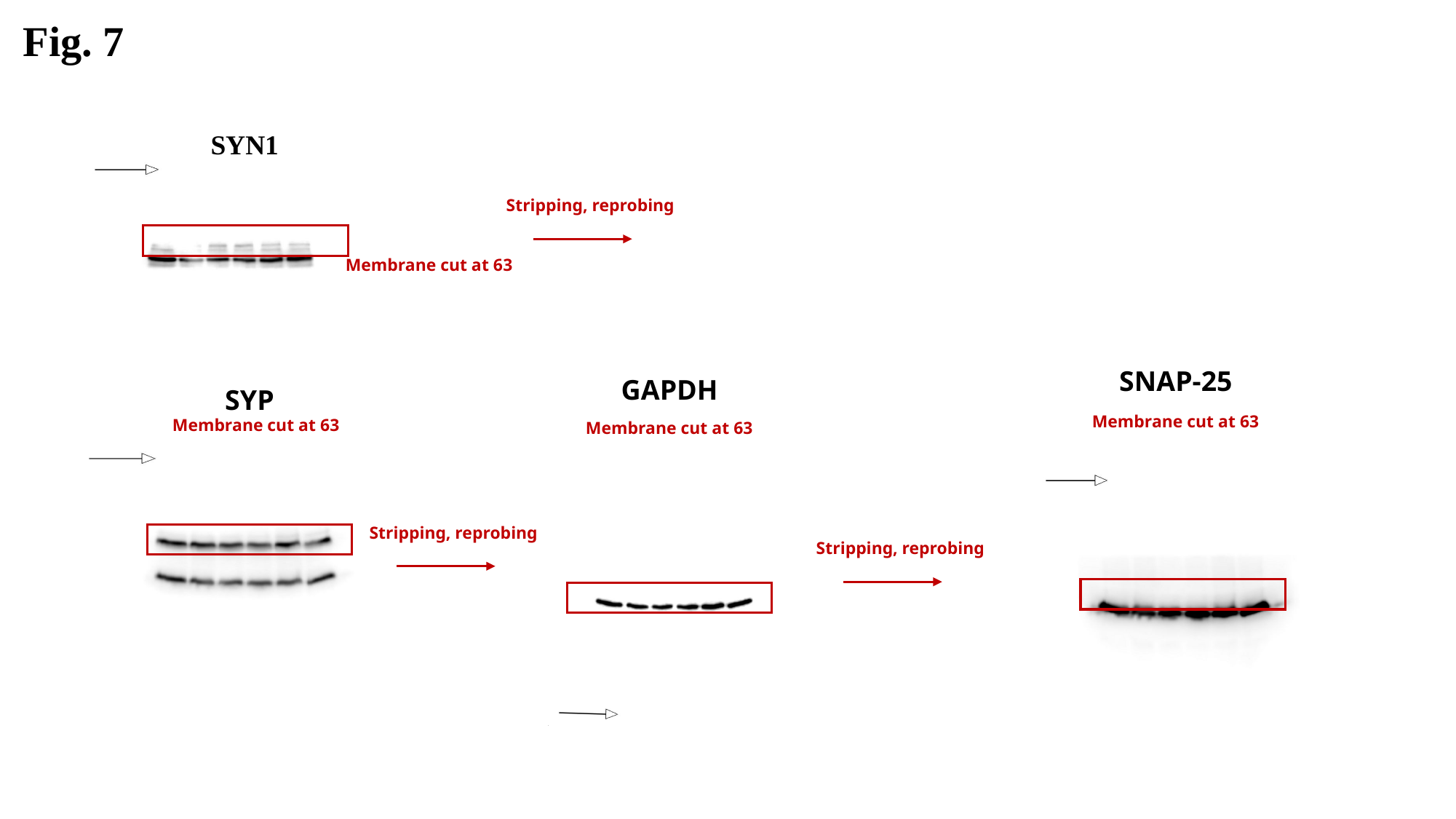

Fig. 7
SYN1
Stripping, reprobing
Membrane cut at 63
SNAP-25
GAPDH
SYP
Membrane cut at 63
Membrane cut at 63
Membrane cut at 63
Stripping, reprobing
Stripping, reprobing

## Slide 12
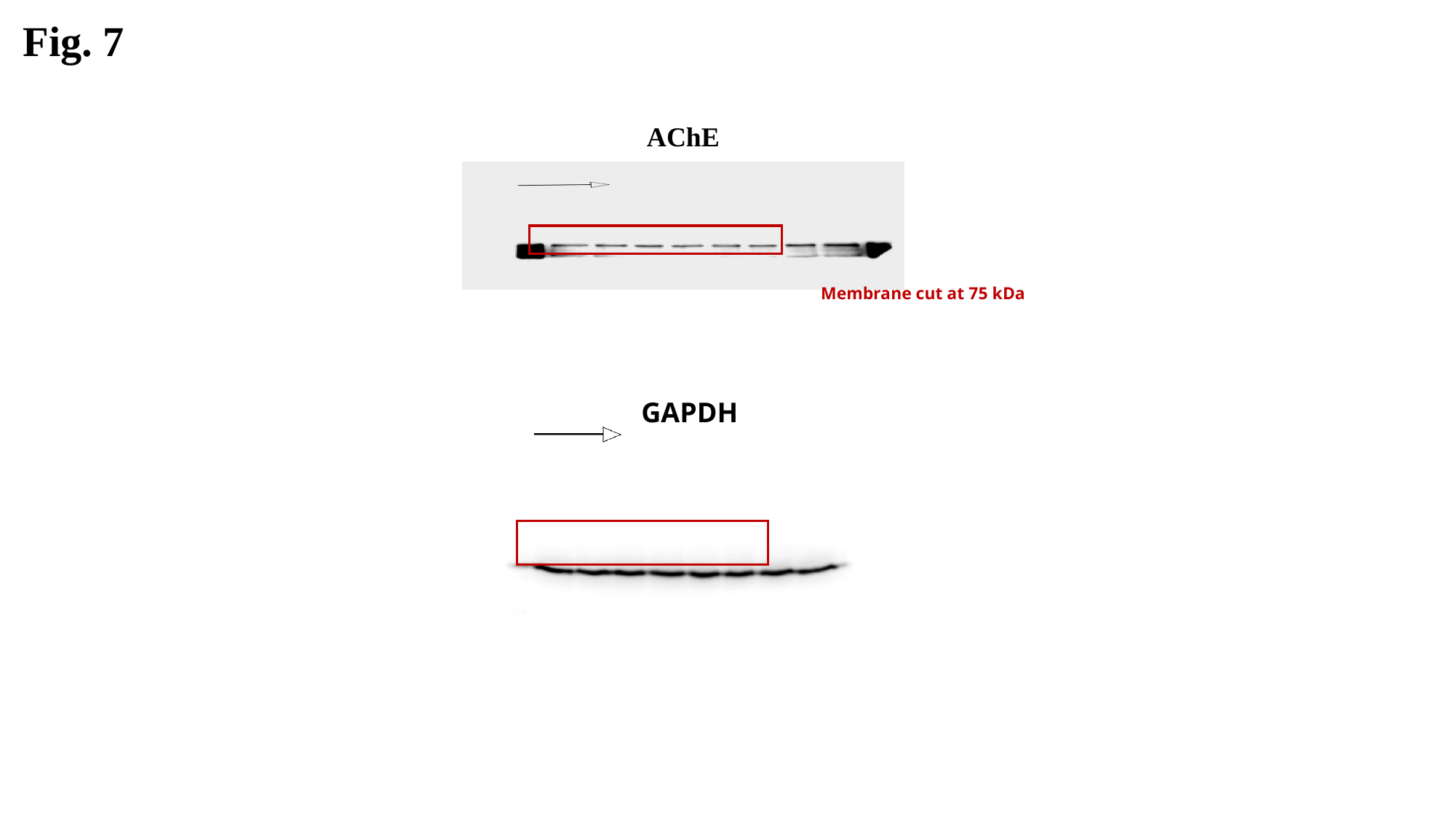

Fig. 7
AChE
Membrane cut at 75 kDa
GAPDH

## Slide 13
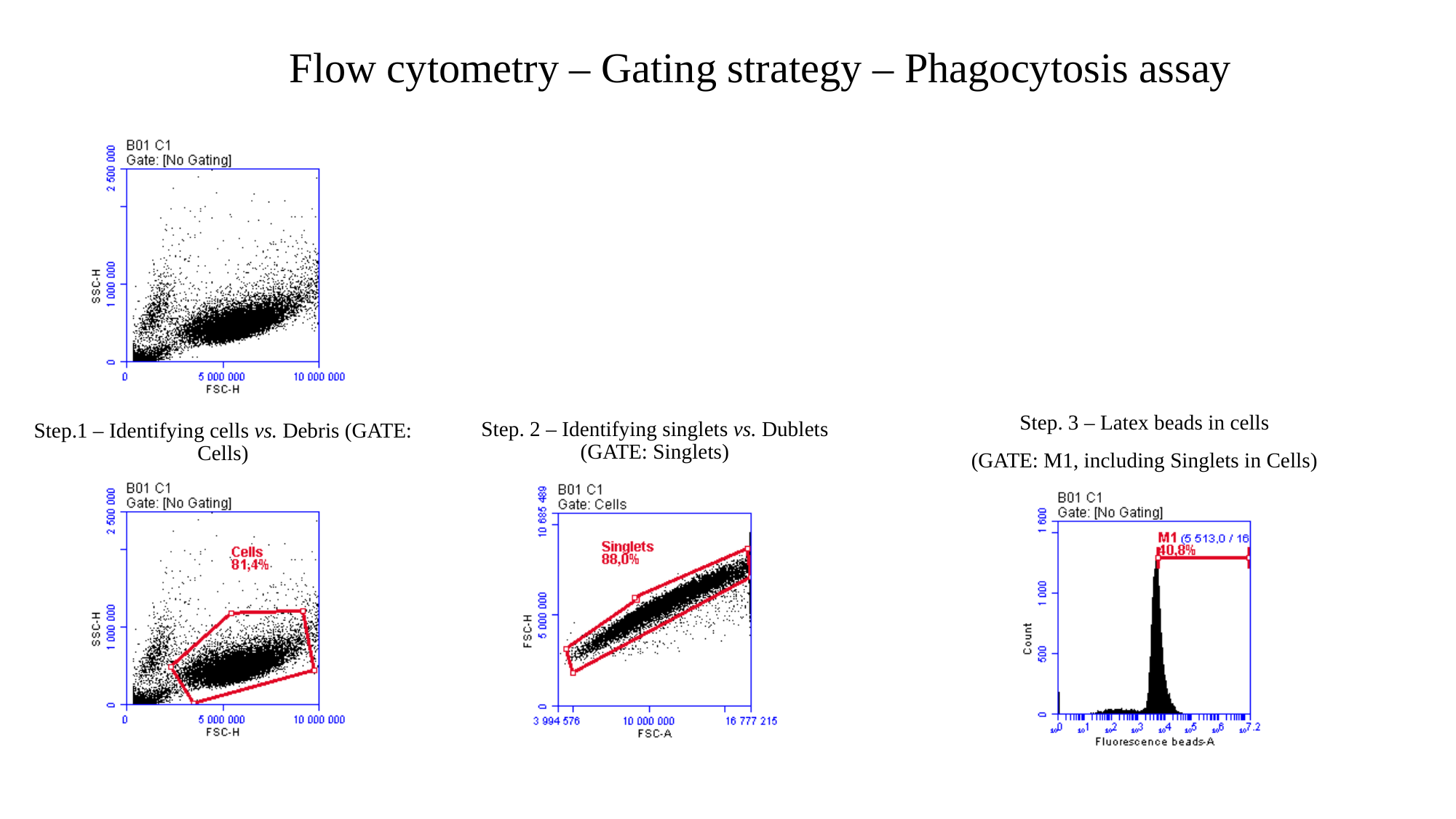

Flow cytometry – Gating strategy – Phagocytosis assay
Step. 2 – Identifying singlets vs. Dublets (GATE: Singlets)
Step.1 – Identifying cells vs. Debris (GATE: Cells)
Step. 3 – Latex beads in cells
(GATE: M1, including Singlets in Cells)
